# A Protein Antagonist of Activation-Induced Cytidine Deaminase Encoded by a Complex Mouse Retrovirus

**DOI:** 10.1128/mBio.01678-19

**Published:** 2019-08-13

**Authors:** Gurvani B. Singh, Hyewon Byun, Almas F. Ali, Frank Medina, Dennis Wylie, Haridha Shivram, Andrea K. Nash, Mary M. Lozano, Jaquelin P. Dudley

**Affiliations:** a Dept. of Molecular Biosciences, LaMontagne Center for Infectious Disease, and Institute for Cellular and Molecular Biology, The University of Texas at Austin, Austin, Texas, USA; b Computational Biology and Bioinformatics and Center for Biomedical Research Support, The University of Texas at Austin, Austin, Texas, USA; University of Washington; Boston College; University of Chicago

**Keywords:** AID inhibitor, Apobec3, activation-induced cytidine deaminase, mouse mammary tumor virus, retroviruses

## Abstract

Complex human-pathogenic retroviruses cause high morbidity and mortality worldwide, but resist antiviral drugs and vaccine development due to evasion of the immune response. A complex retrovirus, mouse mammary tumor virus (MMTV), requires replication in B and T lymphocytes for mammary gland transmission and is antagonized by the innate immune restriction factor murine Apobec3 (mA3). To determine whether the regulatory/accessory protein Rem affects innate responses to MMTV, a splice-donor mutant (MMTV-SD) lacking Rem expression was injected into BALB/c mice. Mammary tumors induced by MMTV-SD had a lower proviral load, lower incidence, and longer latency than mammary tumors induced by wild-type MMTV (MMTV-WT). MMTV-SD proviruses had many G-to-A mutations on the proviral plus strand, but also C-to-T transitions within WRC motifs. Similarly, a lymphomagenic MMTV variant lacking Rem expression showed decreased proviral loads and increased WRC motif mutations relative to those in wild-type-virus-induced tumors, consistent with activation-induced cytidine deaminase (AID) mutagenesis in lymphoid cells. These mutations are typical of the Apobec family member AID, a B-cell-specific mutagenic protein involved in antibody variable region hypermutation. In contrast, mutations in WRC motifs and proviral loads were similar in MMTV-WT and MMTV-SD proviruses from tumors in AID-insufficient mice. AID was not packaged in MMTV virions. Rem coexpression in transfection experiments led to AID proteasomal degradation. Our data suggest that *rem* specifies a human-pathogenic immunodeficiency virus type 1 (HIV-1) Vif-like protein that inhibits AID and antagonizes innate immunity during MMTV replication in lymphocytes.

## Abstract (precis)

Complex retroviruses, such as human-pathogenic immunodeficiency virus type 1 (HIV-1), cause many human deaths. These retroviruses produce lifelong infections through viral proteins that interfere with host immunity. The complex retrovirus mouse mammary tumor virus (MMTV) allows for studies of host-pathogen interactions not possible in humans. A mutation preventing expression of the MMTV Rem protein in two different MMTV strains decreased proviral loads in tumors and increased viral genome mutations typical of an evolutionarily ancient enzyme, AID. Although the presence of AID generally improves antibody-based immunity, it may contribute to human cancer progression. We observed that coexpression of MMTV Rem and AID led to AID destruction. Our results suggest that Rem is the first known protein inhibitor of AID and that further experiments could lead to new disease treatments.

## INTRODUCTION

**FIG 1:**
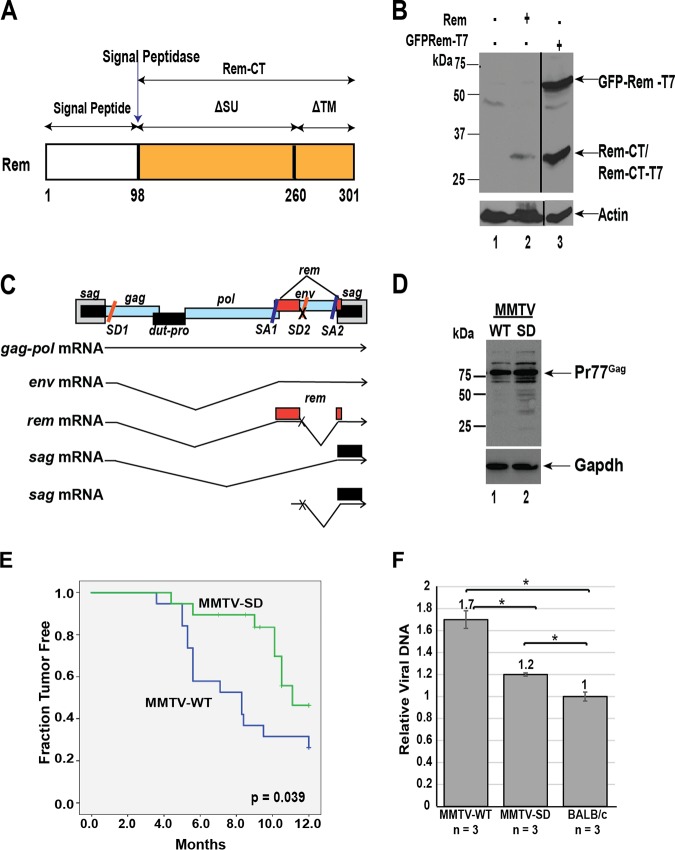
MMTVs lacking Rem expression have a reduced incidence and increased latency of mammary tumors. (A) Diagram of the Rem precursor and the cleavage products, SP and Rem-CT. Rem is synthesized as a precursor that is cleaved by signal peptidase at the ER membrane into SP (white box) and Rem-CT (yellow box). The ΔSU and ΔTM designations have been used to indicate that Rem represents an in-frame deletion of the Env protein. Env is translated from a singly spliced MMTV mRNA, whereas Rem is translated from a doubly spliced viral mRNA. (B) Western blotting of transfected 293T cell extracts with Rem-CT-specific antibody. Positions of the tagged Rem precursor and cleaved Rem-CT (tagged and untagged) are shown in the upper panel. The tagged precursor has GFP on the N terminus and a T7 tag on the C terminus. The bottom panel shows the same extracts with actin-specific antibody. (C) Strategy for generation of Rem-null MMTV and TBLV proviruses. The thick gray boxes represent long terminal repeats (LTRs); the thinner boxes show open reading frames. The Rem coding region is shown in red. The SD mutation (designated by an X) eliminates the downstream SD site needed to generate the doubly spliced *rem* mRNA and the singly spliced *sag* mRNA from the internal *env* promoter. (D) MMTV-WT and MMTV-SD produce equivalent amounts of Gag in tissue culture cells. Stably transfected XC rat cells expressing MMTV-WT or MMTV-SD were used to harvest cell extracts. Western blots were incubated with CA-specific (upper panel) or GAPDH-specific (lower panel) antibody. Western blotting with CA-specific antibody showed similar amounts of Gag precursor (Pr77) expression in cell extracts. (E) BALB/c mice develop mammary tumors with lower incidence and increased latency after infection with the Rem-null (MMTV-SD) virus. Kaplan-Meier plots reveal a difference in mammary tumor development between mice infected with MMTV-WT (blue line) and those infected with MMTV-SD (green line) (see *P* value). (F) MMTV-SD-induced mammary tumors have reduced proviral DNA levels relative to MMTV-WT-induced tumors. PCRs were performed using DNA from three mammary tumors derived by inoculation of three independent BALB/c mice with either MMTV-WT or MMTV-SD, and the results were compared with those using uninfected BALB/c DNA containing endogenous *Mtv* proviruses. Statistical significance of data from comparisons between columns is indicated by an asterisk (*P* < 0.05).

Mouse mammary tumor virus (MMTV) is the only known murine retrovirus that has a complex organization typical of human-pathogenic retroviruses (1) but is amenable to studies of viral interactions with the natural host immune system (2). Like human-pathogenic retroviruses, MMTV has both accessory and regulatory genes (1, 3, 4). MMTV-encoded Rem protein shares some functions with human immunodeficiency virus (HIV) Rev regulatory protein (1, 5), and yet Rem is synthesized as a precursor at the endoplasmic reticulum (ER) membrane (6). Unlike Rev, Rem cleavage by a signal peptidase generates an N-terminal signal peptide (SP) and a C-terminal protein (Rem-CT) (Fig. 1A) (6). MMTV Rem proteins and envelope (Env) proteins are translated in the same open reading frame and cleaved to yield SP, which is extracted from the endoplasmic reticulum (ER) membrane to serve a Rev-like function (1, 6–8). The function of Rem-CT has not been described.

Rem is synthesized from a doubly spliced viral RNA (1, 5). Loss of the downstream splice donor (SD2) site on viral genomic RNA eliminates *rem* mRNA synthesis as well as production of singly spliced mRNA for the accessory gene *sag* from the envelope promoter, but not for that from the long terminal repeat (LTR) promoter (9). Mutation of the SD2 site (hereafter called SD) does not affect MMTV replication in cell culture (9). Nevertheless, the SD mutation decreases MMTV replication in lymphoid cells as judged by reduced Sag-specific T-cell deletion and mammary tumorigenesis, suggesting that this splicing event is critical for replication *in vivo* (9). Prior experiments have revealed that MMTV requires replication in both B and T lymphocytes for efficient mammary gland transmission (10, 11). Human APOBEC and murine Apobec family proteins are restriction factors for retroviruses that replicate in T lymphocytes, including HIV-1 and murine leukemia viruses (MuLVs) (12). HIV-1 Vif and MuLV GlycoGag are known antagonists of these cytidine deaminases, acting either by mediating proteasomal degradation to prevent packaging into viral particles or by being incorporated into virions for exclusion of deaminase access to viral cores (13–19). Incorporation of APOBEC/Apobec enzymes into viral particles yields cytidine deamination on the minus strand, leading to G-to-A transition mutations on the plus strand during proviral DNA synthesis or, alternatively, to blocks to reverse transcription. Both processes decrease virus infectivity (20, 21). MMTV infection of mice deficient for murine Apobec3 (mA3) resulted in accelerated mammary tumorigenesis and increased viral loads relative to infection of wild-type C57BL/6 mice (22). Although MMTV requires replication in B and T lymphocytes prior to mammary gland transmission (3, 10, 11), no MMTV-encoded factor that antagonizes mA3 has been identified.

Here we assess the potential role of Rem and its C-terminal cleavage product as accessory factors *in vivo*, particularly with respect to Apobec-mediated restriction of MMTV replication. Inoculation of BALB/c mice independently with two different MMTV strains lacking Rem expression (one Sag-dependent strain and one Sag-independent strain) resulted in tumors with decreased proviral loads and increased hypermutation in both cases. We observed numerous transition mutations on the proviral plus strand, which is not typical of mA3 (23–25). In contrast, an Apobec family member, activation-induced cytidine deaminase (AID), is known to cause hypermutations on both strands of immunoglobulin genes during B-cell differentiation (26, 27). Differences in MMTV proviral load and most proviral mutations were eliminated in AID-deficient (*Aicda*^−/−^) mice lacking murine AID (mAID) expression. Furthermore, Rem coexpression with AID resulted in proteasomal degradation of AID, similarly to the effect of Vif coexpression with human APOBEC3G (A3G) (14–17). Our results strongly suggest that Rem is an antagonist of murine AID (mAID) (28). Although previous data indicate that AID shapes adaptive antibody responses to many pathogens through antibody affinity maturation (29), our data predict that AID contributes to innate immune responses to retroviruses.

## RESULTS

### Reduced incidence and increased latency of mammary tumors induced by Rem-null MMTV.

Rem is a precursor protein that is cleaved by signal peptidase into an N-terminal SP and Rem-CT (Fig. 1A). To confirm the synthesis of the Rem-CT product, we performed transfection experiments in 293T cells with plasmids expressing either untagged or tagged *rem* expression plasmids. Both plasmids produced Rem-CT (Fig. 1B), which has no known function.

To determine Rem-CT activity *in vivo*, we used the previously described infectious MMTV provirus carrying the downstream SD mutation (MMTV-SD) (Fig. 1C). The SD mutation consists of 6 mutations, resulting in a single valine-to-leucine change in the Env protein and no replication defects in tissue culture (9). MMTV requires superantigen (Sag)-mediated amplification in T cells and mature B cells for transmission to the mammary gland prior to breast cancer induction (10, 11, 30). The MMTV-SD mutant was previously shown to produce normal viral RNA levels as well as intracellular Gag levels equivalent to those seen with wild-type MMTV (MMTV-WT) in tissue culture (Fig. 1D) (9). As expected, reverse transcription-PCR (RT-PCR) analyses of spliced viral mRNAs indicated loss of *rem* upon introduction of the SD mutation (see Fig. S1A in the supplemental material). Similar levels of SP activity were produced from Env processing after transfection of mutant and wild-type MMTV proviruses (Fig. S1B), indicating that absence of Rem expression does not affect Env production in cultured fibroblasts. In agreement with previous results (9), the fraction of animals with mammary tumors decreased and levels of tumor latency increased after inoculation of MMTV-SD compared to MMTV-WT (Fig. 1E). To determine if the SD mutation affected proviral copy numbers in tumors, proviral loads were assessed by PCR. MMTV-WT-induced or MMTV-SD-induced tumors had increased loads compared to the levels of DNA from uninfected mice containing copies of endogenous *Mtv*s (Fig. 1F), suggesting that these tumors had acquired between 4 and 7 haploid proviruses. The average proviral load of three independent tumors from different mice revealed that the loads in tumors induced by MMTV-WT were only slightly higher than, but significantly different from, those in tumors induced by MMTV-SD.

10.1128/mBio.01678-19.1 FIG S1 Rem-null MMTV proviruses lack *rem* mRNA synthesis and retain SP activity. (A) RNA from XC fibroblast cells stably expressing either MMTV-WT or MMTV-SD and from untransfected cells was used for RT-PCR analyses. Primers were designed for the MMTV LTR (upper panel) or the *Gapdh* gene (lower panel). (B) Transient transfections of MMTV-WT and MMTV-SD in XC rat fibroblasts. Cells were transfected and incubated in the presence or absence of dexamethasone (Dex) as indicated to stimulate the hormone-inducible MMTV LTR. SP activity as measured by a *Renilla* luciferase reporter assay is preserved by the Rem-null MMTV provirus. NS, not significant. Transfection of a CMV promoter-driven Rem expression plasmid was used as a positive control. The majority of SP activity was derived from Env cleavage, with results indicating no difference in Env production levels between MMTV-WT and MMTV-SD. Download FIG S1, TIF file, 0.6 MB. Copyright © 2019 Singh et al. 2019 Singh et al. This content is distributed under the terms of the Creative Commons Attribution 4.0 International license.

### Increased Apobec-mediated mutations in MMTV proviruses lacking Rem expression.

Apobec family proteins are known restriction factors for MMTV replication (22). In specific retroviruses, Apobec-mediated cytidine deaminations lead to G-to-A transition mutations on the plus strand of proviral DNA (23, 31). We considered whether Rem was an antagonist of Apobec enzymes and whether the decreased proviral load in MMTV-SD-induced tumors relative to that of MMTV-WT-induced tumors was the result of Apobec-mediated hypermutation. Therefore, we used mammary tumors from five different mice to extract DNA for mutational analysis by PCR of the *env* region followed by cloning and Sanger sequencing. This method allowed us to obtain a longer region from independent proviral integrations, to avoid the necessity of analysis of the related endogenous *Mtv*s, and to verify whether the original SD mutation was maintained. We selected ∼5 to 6 clones from each tumor based on calculations of proviral loads and eliminated clones with the same mutations to avoid multiple samplings of the same proviruses. The combined plus-strand sequencing results from all proviruses indicated increases in transition and transversion mutations within MMTV-SD proviruses compared to MMTV-WT proviruses, with the largest difference in C-to-T transitions seen on the plus strand (Table 1). Such mutations are typical of Apobec family members (12), although C-to-T mutations induced by mA3 during reverse transcription are expected to occur on the proviral minus strand (12, 17). Further, both transition mutations and transversion mutations were elevated on the plus strand of MMTV-SD proviruses relative to MMTV-WT proviruses (Table 1). Together, these results indicate that alteration of *rem* and/or *sag* mRNA synthesis as a consequence of the SD mutation (i) reduced the ability of MMTV to induce mammary tumors, (ii) decreased the proviral load, and (iii) diminished the genomic integrity of proviruses in these tumors.

**TABLE 1:** Proviral mutation frequency in BALB/c mammary tumors induced by MMTV-WT and MMTV-SD

Mutation type	Mutation frequency^*a*^		Fold increase (SD/WT)
	MMTV-WT	MMTV-SD	
G to A	0.36	1.28	3.6
A to G	0.56	1.22	2.2
**C to T**	**0.08**	**0.66**	**8.3**
T to C	1.08	1.38	1.3
Transitions	2.08	4.54	2.2
Transversions	0.56	1.00	1.8
**WRC**	**0.12**	**0.59**	**4.9**
SYC	0.08	0.28	3.5
TYC	0.00	0.53	–
ATC	0.12	0.19	1.6

a Number of mutations/number of clones was determined on the basis of Sanger sequencing of 1,100 bp of the plus strand of at least five proviral *env* gene clones obtained from each of five independent BALB/c tumors induced by MMTV-WT (*n* = 25) or MMTV-SD (*n* = 32). Frequencies of WRC, SYC, TYC, and ATC-motif mutations were calculated from both strands. W = A or T; R = A or G; S = G or C; Y = C or T. For statistical analysis of the data, see scatter plots in Fig. 2. Boldface data highlight the greatest differences between MMTV-WT and MMTV-SD sequences typical of AID-mediated mutagenesis.

Next, proviral sequences were analyzed for mutations occurring in consensus sites that are preferentially targeted by different Apobec family members. Apobec3 is known to induce G-to-A hypermutation on the retroviral plus strand of some MuLVs during reverse transcription in lymphocytes (23, 32). However, the Apobec family member AID acts on both host DNA strands, leading to G-to-A and C-to-T mutations on the coding strand of the variable region of the immunoglobulin genes (28). AID-induced immunoglobulin gene mutations often are followed by repair of deaminated cytidines by an error-prone process (33, 34), resulting in additional mutations. AID activity also results in class switch recombination (28) and, in some cases, cancer (29). This cytidine deaminase is preferentially expressed in germinal center B cells (35), and MMTV requires replication in mature B cells for efficient mammary gland transmission (10). Interestingly, a clear selection was observed for SD site repair since 22% of the MMTV-SD proviruses isolated from mammary tumors contained a wild-type splice site, presumably resulting from recombination with endogenous *Mtv*s, since all 6 bp of the mutant SD site sequence were restored. Therefore, we explored whether the increased levels of mutations observed in MMTV-SD proviruses that retained the SD site mutation (MMTV-SD non-recombinants) and in those that had repaired the SD site mutation (MMTV-SD recombinants) were consistent with preferred AID and mA3 motifs.

Preliminary examination of motifs associated with Apobec-mediated mutagenesis revealed differences between MMTV-WT and MMTV-SD proviruses (Table 1). Since the variation in the number of mutations among clones was large, we applied a nonparametric statistical test to examine significant differences in the distributions of the numbers of mutations/clone. Mutations observed in MMTV proviruses were analyzed for the WRC motif, reported to be a “hot spot” for AID-induced base changes within immunoglobulin genes (36). The distribution of clones with cytidine mutations in the WRC context was not significantly different between MMTV-WT and MMTV-SD nonrecombinant proviruses (Fig. 2A). However, the clones carrying the splice site reversion (MMTV-SD recombinants), which would allow normal *sag* and *rem* mRNA splicing (1, 5), had an increased number of cytidine changes compared with either the MMTV-WT or MMTV-SD nonrecombinant proviruses. In contrast, the distribution of cytidine mutations in the SYC context, which has been described previously as an AID “cold spot” (37, 38), was not significantly different across any of the three groups of proviruses (Fig. 2B). We also analyzed cytidine changes in two other sequence contexts (TYC and ATC). Mutations in TYC motifs are typical of mA3 (23, 39), but changes in the ATC motif were detected by our TransitionFinder software. Analysis of the distribution of TYC mutations/clone indicated a significant difference in the MMTV-SD proviruses regardless of the splice donor site reversion (Fig. 2C). Mutations in the ATC context have also been associated with mA3 *in vitro* (40), and analysis of the distribution of mutations/clone indicated a significant enrichment only in the MMTV-SD recombinant proviruses (Fig. 2D). Interestingly, the MMTV-SD recombinant proviruses, which correct the SD mutation by recombination with *Mtv*s, have multiple base changes, but lack stop codons in the envelope region (Fig. S2), suggesting selection for replication-competent viruses after Apobec-mediated mutagenesis.

**FIG 2:**
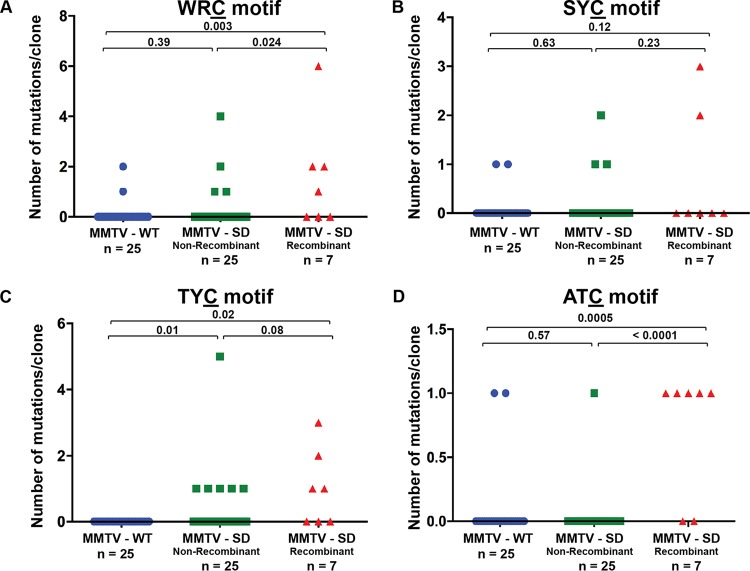
Mutational analysis of proviral envelope genes from BALB/c mammary tumors induced by MMTV-WT or MMTV-SD (Rem-null). The number of C mutations within different motifs on either proviral strand is presented graphically for each clone. The number of clones (n) is indicated. Sequences were obtained from independent clones from five tumors in different animals. MMTV-SD proviruses recovered from mammary tumors were classified as non-recombinant (retaining the inoculated MMTV-SD sequence) or recombinant (carrying the wild-type SD2 sequence after recombination with endogenous *Mtv*s). (A) The number of mutations/clone in the WRC motif typical of AID mutation hot spots. (B) The number of mutations/clone in the SYC motif. (C) The number of mutations/clone in the TYC motif typical of mA3 mutation hot spots. (D) The number of mutations/clone in the ATC motif. Statistical significance of results of nonparametric Mann-Whitney tests is indicated on the scatter plots. The mutant C residue in each motif is underlined.

10.1128/mBio.01678-19.2 FIG S2 MMTV-SD recombinant proviruses lack stop codons. Amino acid differences between the MMTV-WT and MMTV-SD recombinant proviruses in the *env* gene were determined for individual clones by Sanger sequencing. Proviral gene mutations in the absence of Rem yielded recombinants that lack stop codons within the *env* open reading frame of the MMTV-SD proviruses. Download FIG S2, JPG file, 2.0 MB. Copyright © 2019 Singh et al. 2019 Singh et al. This content is distributed under the terms of the Creative Commons Attribution 4.0 International license.

### Increased Apobec-mediated mutations in Sag-independent MMTV (TBLV) lacking Rem expression.

**FIG 3:**
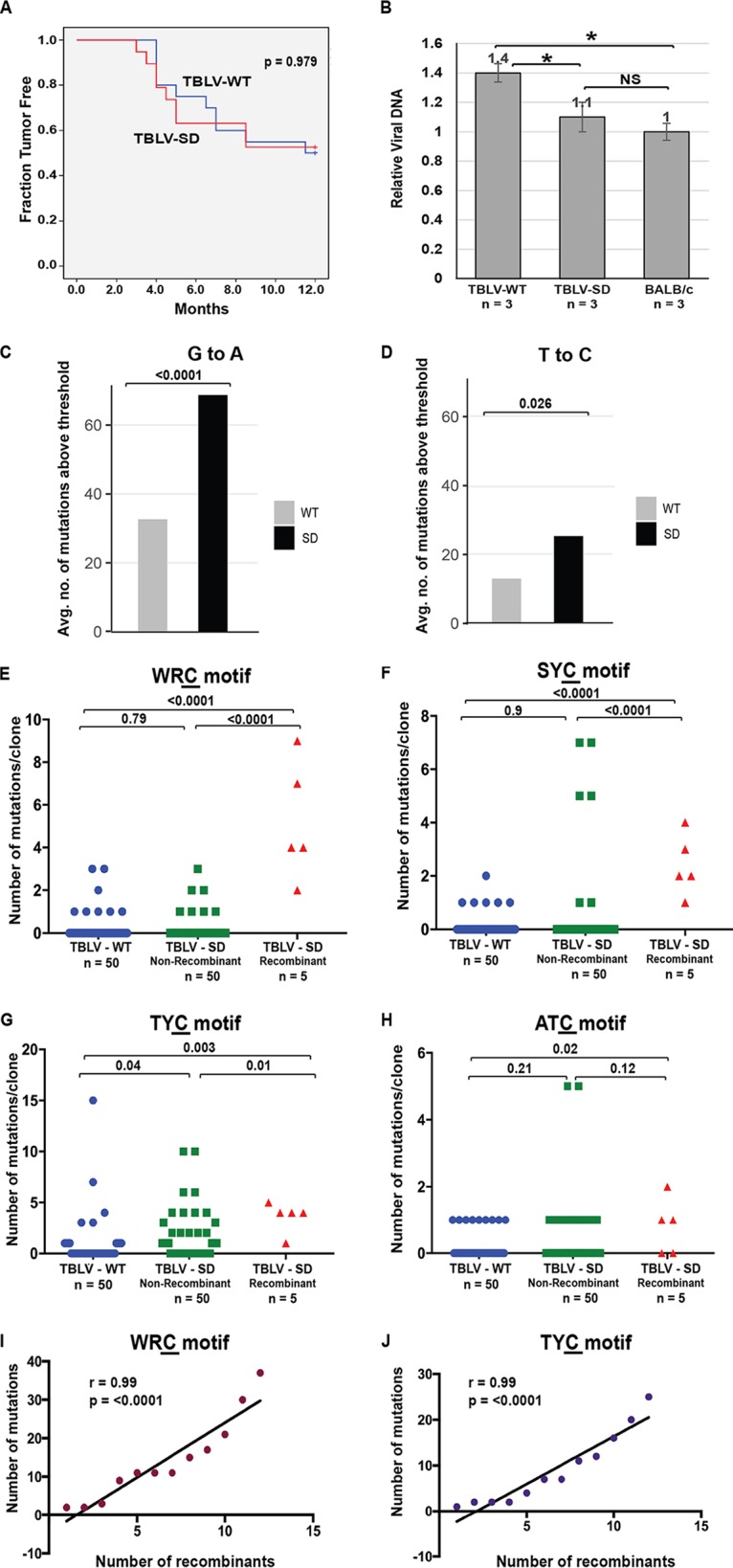
Mutational analysis of proviral envelope gene from BALB/c thymic tumors induced by TBLV-WT or TBLV-SD (Rem-null). (A) Kaplan-Meier plots for thymic tumor development in BALB/c mice injected with TBLV-WT (blue line) or TBLV-SD (red line) are shown with *P* value. (B) TBLV-SD-induced thymic tumors have reduced viral DNA levels relative to TBLV-WT-induced tumors. PCRs were performed using DNA from three thymic tumors derived by inoculation of three independent BALB/c mice with either TBLV-WT or TBLV-SD, and the results were compared with those from uninfected BALB/c DNA containing endogenous *Mtv* proviruses. Statistical significance of data from comparisons between columns is indicated by an asterisk (*P* < 0.05). NS = not significant. (C) Analysis of the average number of G-to-A mutations above a 3% threshold within proviruses obtained from three independent TBLV-WT-induced or TBLV-SD-induced tumors after PCR and Illumina sequencing. (D) Analysis of the average number of T-to-C mutations above a 3% threshold within proviruses obtained from three independent TBLV-WT-induced or TBLV-SD-induced tumors after PCR and Illumina sequencing. (E to H) Analysis of independent clones obtained after PCR and Sanger sequencing. TBLV-SD proviral clones recovered from T-cell tumors were classified as non-recombinant (retaining the inoculated TBLV-SD sequence) or recombinant (carrying the wild-type SD2 sequence after recombination with endogenous *Mtv*s). The number of C mutations within different motifs on either proviral strand is presented graphically for each clone. The number of clones (n) is indicated. Sequences were obtained from independent clones from three tumors in different animals. (E) The number of mutations/clone in the WRC motif typical of AID mutation hot spots. (F) The number of mutations/clone in the SYC motif. (G) The number of mutations/clone in the TYC motif typical of mA3 mutation hot spots. (H) The number of mutations/clone in the ATC motif. Statistical significance of results of nonparametric Mann-Whitney tests is indicated on the scatter plots. The mutant C residue in each motif is underlined. (I and J) Spearman’s correlation coefficient (r) indicating high correlation between number of recombinants and number of mutations in WRC (I) and TYC (J) motifs. Correlation coefficient *P* values are shown on the graph.

To address specifically whether MMTV-encoded Rem or Sag accounts for differences in Apobec-induced mutations in tumor-derived proviruses, we used the MMTV-related retrovirus, type B leukemogenic virus (TBLV). TBLV encodes a truncated, non-functional *sag* gene and lacks transmission to the mammary gland. Infection with TBLV, which has a T-cell-tropic LTR enhancer, induces lymphomas, rather than breast cancer, by insertional mutagenesis (41–43). The infectious TBLV molecular clone (TBLV-WT) differs from MMTV-WT only within the U3 region and does not require *sag* for replication *in vivo* (41). We introduced the SD mutation into TBLV-WT (TBLV-SD) prior to transfection into human Jurkat T cells, which lack endogenous *Mtv*s. As demonstrated for MMTV, *env* mRNA levels and SP activity remained relatively unaffected by the SD mutation, while *rem* mRNA production was blocked (Fig. S3A and B). No differences in the levels of virus production were observed between cells expressing TBLV-WT and those expressing TBLV-SD (Fig. S3C). In contrast to results with MMTV, injection of TBLV-WT and TBLV-SD into BALB/c mice gave no difference in incidence or latency of thymic lymphomas (Fig. 3A), although, like MMTV-induced tumors, we observed statistically significant differences in proviral loads (Fig. 3B). These results suggest that the altered tumor incidence and latency seen with the mice inoculated with MMTV-WT relative to those inoculated with MMTV-SD were due to the differences in the levels of *sag* mRNA expression from the intragenic *env* promoter (44) as originally reported (9). Both the MMTV-SD-induced tumors and the TBLV-SD-induced tumors had lower proviral loads than their wild-type counterparts, suggesting that Rem C-terminal sequences may counteract factors that limit virus replication in BALB/c mice. Since TBLV does not replicate in the mammary gland, restriction appears to occur at an early step after viral infection, such as during replication in B and T cells, prior to virus transmission to the mammary gland (2).

10.1128/mBio.01678-19.3 FIG S3 TBLV-SD proviruses retain SP activity and produce equivalent amounts of virus in tissue culture cells. (A) RNA from human Jurkat cells stably expressing either TBLV-WT or TBLV-SD and from untransfected cells was used for RT-PCRs. Primers were designed for the TBLV LTR (upper panel) or the *GAPDH* gene (lower panel). (B) Transient transfections of TBLV-WT and TBLV-SD in human Jurkat cells. The SP activity of TBLV-SD proviruses (as measured by a *Renilla* luciferase reporter assay) was preserved. SP was synthesized primarily from *env* mRNA, which was more abundant than *rem* mRNA. Transfection of a CMV-promoter-driven Rem expression plasmid was used as a positive control. The asterisk indicates significance (*P* < 0.05). (C) Stably transfected Jurkat cells expressing TBLV-WT or TBLV-SD were used to harvest supernatants or for preparation of cytosolic extracts. Western blotting showed similar amounts of Gag expression in whole-cell lysates and released virus in supernatants from cells transfected with expression plasmids for TBLV-WT or TBLV-SD. Western blots were incubated with CA-specific or actin-specific antibody. Arrows indicate the positions of cleaved capsid or capsid precursor (Pr77^Gag^). Download FIG S3, TIF file, 1.4 MB. Copyright © 2019 Singh et al. 2019 Singh et al. This content is distributed under the terms of the Creative Commons Attribution 4.0 International license.

To test whether TBLV-SD proviruses also showed evidence of Apobec-mediated mutagenesis, independent T-cell lymphomas from three different wild-type TBLV-infected or SD mutant-infected mice were used for PCR amplification of two different proviral regions (the envelope-3′ LTR and polymerase regions). Because TBLV induces polyclonal tumors (45), unlike the more clonal MMTV-induced mammary cancers (46), high-throughput sequencing was used initially for the analysis of proviral mutations. We mapped proviral paired-end reads from both TBLV-WT-induced and TBLV-SD-induced tumors to the original sequence of the cloned provirus. After subtracting a 3% error rate for each base due to PCR effects, data from averaged reads indicated SD site reversion within some proviruses in the mutant-induced tumors relative to TBLV-WT-induced tumors, consistent with selection to maintain Rem expression (see Table S1 in the supplemental material).

10.1128/mBio.01678-19.9 TABLE S1 Reversion of the SD site in TBLV-SD proviruses detected by Illumina sequencing. Download Table S1, DOCX file, 0.02 MB. Copyright © 2019 Singh et al. 2019 Singh et al. This content is distributed under the terms of the Creative Commons Attribution 4.0 International license.

To assess Apobec-induced mutations in proviruses from TBLV-SD-induced tumors compared to those from TBLV-WT-induced tumors, we eliminated likely PCR duplicates and quantified the number of reads aligned with each base compared to the reference sequence. The dichotomized alternative base frequency was then modeled as a function of sample group (WT or SD) using a mixed-effects logistical regression approach incorporating a random effect accounting for intersample variation within the group. The G-to-A changes on the plus strand within TBLV-SD proviruses were highly statistically significantly different from those observed within TBLV-WT proviruses (Fig. 3C), in agreement with the mA3-induced changes (12, 25, 32). We also observed increased A-to-G, C-to-T, and T-to-C transitions on the plus strand of TBLV-SD proviruses relative to TBLV-WT proviruses, which are not typical of mA3 (25, 32) (Fig. 3D; see also Fig. S4). Differences were not observed between *Gapdh* gene sequences obtained from TBLV-WT-induced and SD-induced tumors after analysis by the same method (Fig. S4). Since TBLV does not induce mammary tumors (47–49), these data were consistent with generation of both mA3 mutations and non-mA3 mutations during viral replication in hematopoietic cells.

10.1128/mBio.01678-19.4 FIG S4 Analysis of high-throughput data obtained from Illumina sequencing of products obtained by PCR of TBLV-induced tumor DNA. Reads were analyzed from tumor-derived TBLV-WT proviruses (gray columns) or TBLV-SD proviruses (black columns) (each obtained from three independent tumors as described in Materials and Methods). The TBLV data were obtained from a mixture of PCR products derived from the *pol*, *env*, and 3′ LTR regions on the proviral plus strand. For each reference base of TBLV (upper panels) or *Gapdh* (lower panels), the alternative base is given on the *x* axis. None of the *Gapdh* changes were significantly different between the TBLV-WT and TBLV-SD proviruses. G-to-A changes were highly statistically significantly different (*P* = 6.3 × 10^−8^) between the TBLV-WT and TBLV-SD proviruses, but T-to-C transitions also were statistically significantly different (*P* = 0.026). Although TBLV-SD proviruses tended to have greater numbers of other transition and transversion mutations, these changes were not significantly different by the mixed-effect logistic regression model. (A) Data averaged for proviruses from all three TBLV-WT and TBLV-SD tumors. (B) Data for proviruses from individual tumors. Download FIG S4, TIF file, 1.0 MB. Copyright © 2019 Singh et al. 2019 Singh et al. This content is distributed under the terms of the Creative Commons Attribution 4.0 International license.

To analyze longer regions for detection of Apobec-mediated consensus site mutations present only within the envelope gene, DNA samples from three different polyclonal TBLV-induced T-cell lymphomas were used for PCR. Individual clones then were subjected to Sanger sequencing. Analysis of >50 *env* clones from either the TBLV-WT-induced or TBLV-SD-induced tumors revealed increased levels of transition mutations in acquired proviruses, with the highest increase in C-to-T mutations on the plus strand (Table 2). These data were slightly different from the high-throughput results since the latter data included the *pol*, *env*, and 3′ LTR regions and did not distinguish the 9% of the TBLV-SD clones with SD site reversion by recombination with endogenous *Mtv*s. Previous results showed that the levels of human A3G mutations are highest for single-stranded DNA mutagenesis just 5′ to the polypurine tracts, a region targeted by our Sanger sequencing (50). Moreover, four out of five recombinants (80%) had stop codons in the *env* gene (Fig. S5). As a control, Sanger sequencing of multiple clones of the c-*Myc* gene were analyzed, and no difference was observed in the number of sequence changes between clones from TBLV-WT-induced and TBLV-SD-induced tumors (Table S2). These results suggested that, in contrast to MMTV-SD recombinants, little selective pressure was applied to maintain the integrity of TBLV proviruses after *Mtv* recombination, which presumably occurred in lymphoid cells.

**TABLE 2:** Proviral mutation frequency in BALB/c T-cell lymphomas induced by TBLV-WT and TBLV-SD

Mutation type	Mutation frequency^*a*^		Fold increase (SD/WT)
	TBLV-WT	TBLV-SD	
G to A	2.02	2.98	1.5
A to G	0.60	1.27	2.1
**C to T**	**0.08**	**0.73**	**9.1**
T to C	0.86	1.53	1.8
Transitions	3.56	6.51	1.8
Transversions	0.10	0.71	7.1
**WRC**	**0.26**	**0.67**	**2.6**
SYC	0.14	0.69	4.9
TYC	0.84	1.65	2.0
ATC	0.18	0.47	2.6

a Number of mutations/number of clones was determined on the basis of Sanger sequencing of 1,100 bp of the plus strand of at least fifteen proviral *env* gene clones obtained from each of three independent BALB/c tumors induced by TBLV-WT (*n* = 50) or TBLV-SD (*n* = 55). Frequencies of WRC, SYC, TYC, and ATC-motif mutations were calculated from both strands. W = A or T; R = A or G; S = G or C; Y = C or T. For statistical analysis of the data, see scatter plots in Fig. 3. Boldface data highlight the greatest differences between MMTV-WT and MMTV-SD sequences typical of AID-mediated mutagenesis.

10.1128/mBio.01678-19.5 FIG S5 TBLV-SD revertant proviruses have stop codons. Amino acid differences between TBLV-WT and TBLV-SD recombinant proviruses in the *env* gene were determined for individual clones by Sanger sequencing. Unlike MMTV-SD recombinants, increased viral gene mutations in the absence of Rem resulted in increased numbers of stop codons within the *env* open reading frame of the TBLV-SD recombinant proviruses. Stop codons are shown with an asterisk and are boxed in red. Download FIG S5, JPG file, 1.6 MB. Copyright © 2019 Singh et al. 2019 Singh et al. This content is distributed under the terms of the Creative Commons Attribution 4.0 International license.

10.1128/mBio.01678-19.10 TABLE S2 Mutation frequency in the *c-Myc* gene from TBLV-WT and TBLV-SD proviruses from thymic tumors determined by Sanger sequencing. Download Table S2, DOCX file, 0.01 MB. Copyright © 2019 Singh et al. 2019 Singh et al. This content is distributed under the terms of the Creative Commons Attribution 4.0 International license.

We also analyzed the distribution of mutations within specific sequence motifs in TBLV proviruses from T-cell tumors. TBLV-SD proviruses had increased mutations in Apobec-associated sequence motifs (Table 2). Statistical analysis of the distribution of WRC and SYC-motif mutations/clone within indicated a significant increase in the distribution of mutations/clone within TBLV-SD recombinant proviruses compared to TBLV-WT and TBLV-SD non-recombinant proviruses (Fig. 3E and F). The distribution of TYC motif mutations was significantly different in TBLV-SD proviruses regardless of the splice-donor site reversion by recombination (Fig. 3G). Using the same type of analysis, the ATC motif mutations differed between TBLV-WT and TBLV-SD recombinant proviruses (Fig. 3H). We also examined the relationship between recombination-mediated SD site repair and the number of mutations in WRC, SYC, TYC, and ATC motifs observed in MMTV and TBLV proviruses from T-cell tumors (Fig. 3E to H). A statistically significant correlation was observed between SD site repair and mutation in each of these motifs (Fig. 3I and J; see also Fig. S7). These results suggest that viral replication in lymphoid cells allows both recombination with endogenous *Mtv* transcripts to repair the SD mutation as well as Apobec-mediated hypermutation. Together, these data are consistent with multiple Apobec-type mutations typical of AID and mA3, which occur during a common replication step for both TBLV and MMTV in hematopoietic cells (41).

10.1128/mBio.01678-19.7 FIG S7 Correlation analysis of the number of recombinants and sum of mutations in different motifs from MMTV-induced tumors and TBLV-induced tumors from wild-type BALB/c mice. Recombinants that repaired the SD mutations within C residues of the indicated motifs were analyzed. (A to D) Spearman’s correlation coefficient (*r*) data indicating high correlation between the number of recombinants and the sum of mutations in WRC (A), TYC (B), SYC (C), and ATC (D) motifs, respectively, as determined by Sanger sequencing of proviral envelope genes. Correlation coefficient *P* values are shown on the graph. Download FIG S7, TIF file, 0.3 MB. Copyright © 2019 Singh et al. 2019 Singh et al. This content is distributed under the terms of the Creative Commons Attribution 4.0 International license.

### Rem independence of WRC and TYC motif mutations within MMTV proviruses in AID-deficient mice.

**FIG 4:**
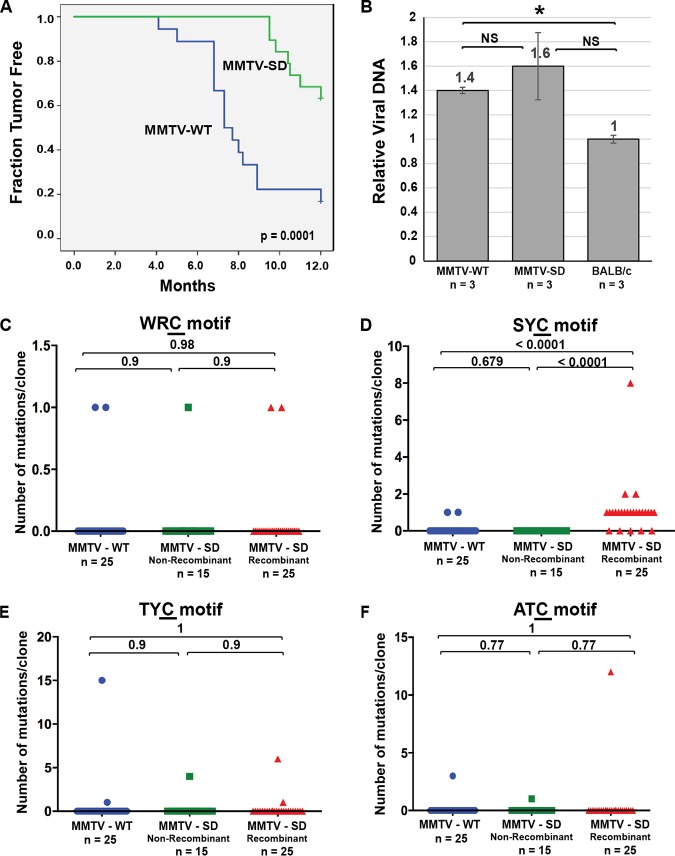
Mutational analysis of the proviral envelope gene from *Aicda*^−/−^ BALB/c mammary tumors induced by MMTV-WT or MMTV-SD (Rem-null). (A) Kaplan-Meier plots for mammary tumor development in *Aicda*^−/−^ BALB/c mice injected with MMTV-WT (blue line) or MMTV-SD (green line) are shown with *P* value. (B) Proviral loads in MMTV-WT-induced and MMTV-SD-induced mammary tumors relative to endogenous *Mtv* proviruses in uninfected *Aicda*^−/−^ mice. Values for three independent tumors from different animals were determined as described for Fig. 1F. Although the values were not significantly different (NS), the trend indicated that MMTV-SD proviral loads were higher than the endogenous *Mtv* levels. (C to F) MMTV-SD proviruses recovered from mammary tumors were classified as non-recombinant (retaining the inoculated MMTV-SD sequence) or recombinant (carrying the wild-type SD2 sequence after recombination with endogenous *Mtv*s). The number of C mutations within different motifs on either proviral strand is presented graphically for each clone. Clone numbers (n) are indicated. Sequences were obtained from independent clones from five tumors in different animals after PCR and Sanger sequencing. (C) The number of mutations/clone in the WRC motif typical of AID mutation hot spots. (D) The number of mutations/clone in the SYC motif. (E) The number of mutations/clone in the TYC motif typical of mA3 mutation hot spots. (F) The number of mutations/clone in the ATC motif. Statistical significance of results of nonparametric Mann-Whitney tests is indicated on the scatter plots. The mutant C residue in each motif is underlined.

To test whether AID is responsible for increased MMTV-induced mutations in the absence of Rem, we infected AID-deficient (*Aicda*^−/−^) mice on the BALB/c background with MMTV-WT or MMTV-SD. Mammary tumors developed more slowly in the *Aicda*^−/−^ mice after inoculation of MMTV-SD than after inoculation of MMTV-WT (Fig. 4A), but this result was not statistically different from that obtained in wild-type BALB/c (Fig. S6). Nevertheless, we observed that the proviral loads were unchanged between MMTV-WT-induced and MMTV-SD-induced tumors in *Aicda*^−/−^ mice, whereas proviral loads in either MMTV-WT-induced or TBLV-WT-induced tumors differed from those in MMTV-SD-induced or TBLV-SD-induced tumors in wild-type BALB/c mice (Fig. 4B). These results suggested that differences in proviral load were due to AID-mediated mutagenesis in AID-expressing mice.

10.1128/mBio.01678-19.6 FIG S6 Kaplan-Meier survival plots for MMTV-infected mice on the BALB/c background. *Aicda*-sufficient BALB/c mice were infected with MMTV-WT or MMTV-SD (blue or green lines, respectively). *Aicda*-deficient BALB/c mice were infected with MMTV-WT or MMTV-SD (red or purple lines, respectively). No significant differences were observed (*P* values are shown) for MMTV-WT-infected mice (A) or MMTV-SD-infected mice (B) in the presence or absence of AID. Download FIG S6, TIF file, 0.2 MB. Copyright © 2019 Singh et al. 2019 Singh et al. This content is distributed under the terms of the Creative Commons Attribution 4.0 International license.

To determine whether proviral mutagenesis was affected in the absence of AID, we obtained DNA from five independent tumors induced by MMTV-WT and MMTV-SD for Sanger sequence analysis of proviruses after PCR and cloning. We observed that 62.5% of the clones from MMTV-SD proviruses had reversion of the original SD mutation, consistent with recombination with endogenous *Mtv*s in a cell type enriched in *Aicda^−^*^/^*^−^* mice. Although G-to-A mutations showed an increased frequency in MMTV-SD compared to MMTV-WT proviruses in *Aicda*^−/−^ mice, C-to-T mutations did not show an increase (Table 3). Further, the mutation frequencies in the AID-associated WRC motif seen in MMTV-WT and MMTV-SD proviruses did not differ in *Aicda*^−/−^ mice (Table 3). The mutation frequency in the WRC motif of MMTV-SD proviruses also greatly declined in *Aicda*^−/−^ mice compared to wild-type BALB/c mice (compare Table 1 to Table 3). Analysis of the distributions of mutations/clone showed no significant differences between WRC, TYC, or ATC motif mutations among the MMTV-WT and either the MMTV-SD non-recombinant proviruses or the recombinant proviruses in *Aicda*^−/−^ mice (Fig. 4C, E, and F). In contrast to the results from MMTV-infected BALB/c wild-type mice, the number of mutations/clone in SYC sites showed enrichment in MMTV-SD recombinant proviruses (Fig. 4D). The correlation between SYC motif mutations and SD site reversion resulting from recombination with endogenous *Mtv*s was maintained in both BALB/c and *Aicda*^−/−^ mice (Fig. S7 and Fig. S8). Due to a lack of mutations in MMTV-SD recombinants recovered from tumors in *Aicda*^−/−^ mice, correlation coefficients could not be calculated for the WRC, TYC, or ATC motifs (Fig. S8). Together, these results indicated that Rem antagonizes AID and cytidine deaminases that induce SYC, TYC, and ATC motif mutations. Our data suggest that loss of Rem activity is analogous to loss of HIV-1-encoded Vif since absence of Vif expression leads to hypermutation of the viral genome by specific Apobec3 cytidine deaminases (16–19, 51, 52).

**TABLE 3:** Proviral mutation frequency in BALB/c *Aicda*^−/−^ mammary tumors induced by MMTV-WT and MMTV-SD

Mutation type	Mutation frequency^*a*^		Fold increase (SD/WT)
	MMTV-WT	MMTV-SD	
G to A	0.80	1.65	2.1
A to G	0.56	0.75	1.3
**C to T**	**0.12**	**0.10**	**0.8**
T to C	0.64	1.28	2.0
Transitions	2.12	3.78	1.8
Transversions	0.20	0.25	1.3
**WRC**	**0.08**	**0.08**	**1.0**
SYC	0.08	0.73	9.1
TYC	0.64	0.28	0.4
ATC	0.12	0.33	2.8

a Number of mutations/number of clones was determined on the basis of Sanger sequencing of 1,100 bp of the plus strand from at least five proviral *env* gene clones obtained from five independent BALB/c *Aicda*^−/−^ tumors induced by MMTV-WT (*n* = 25) or MMTV-SD (*n* = 40). Frequencies of WRC, SYC, TYC, and ATC-motif mutations were calculated from both strands. W = A or T; R = A or G; S = G or C; Y = C or T. For statistical analysis of the data, see scatter plots in Fig. 4. Boldface data highlight the greatest differences between MMTV-WT and MMTV-SD sequences typical of AID-mediated mutagenesis.

10.1128/mBio.01678-19.8 FIG S8 Correlation analysis of the number of recombinants and sum of mutations in different motifs from MMTV-induced tumors from *Aicda*^−/−^ mice. Recombinants that repaired the SD mutations within C residues of the indicated motifs were analyzed. (A to D) Spearman’s correlation coefficient (*r*) data indicating high correlation between the number of recombinants and the sum of mutations in the WRC (A), TYC (B), SYC (C), and ATC (D) motifs, respectively, as determined by Sanger sequencing of proviral envelope genes. A correlation coefficient *P* value could be calculated only for the SYC motif. Download FIG S8, TIF file, 0.3 MB. Copyright © 2019 Singh et al. 2019 Singh et al. This content is distributed under the terms of the Creative Commons Attribution 4.0 International license.

**FIG 5:**
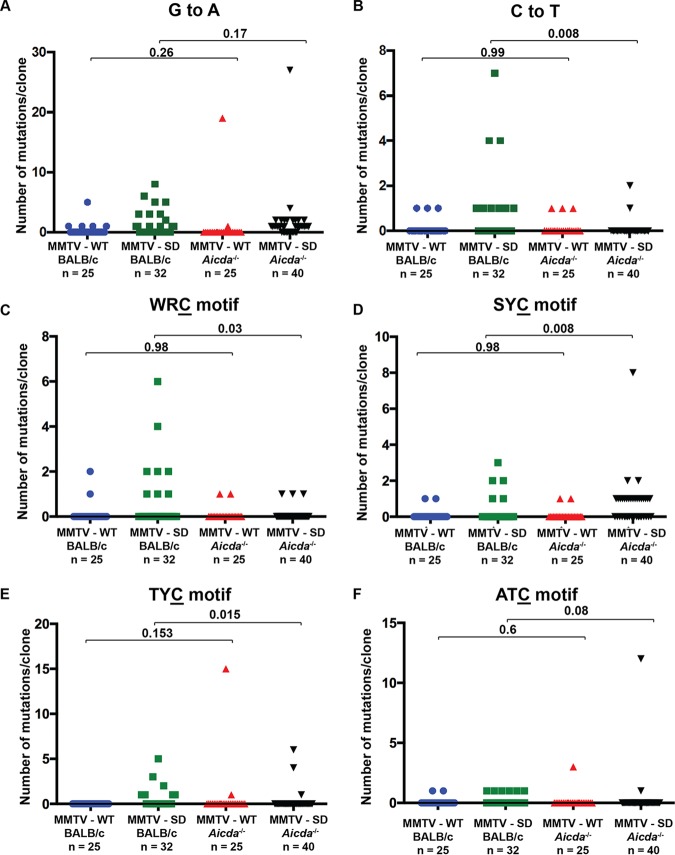
Comparison of distributions of mutations/clone within proviral envelope genes between MMTV-WT-induced and MMTV-SD-induced mammary tumors in either wild-type or *Aicda*^−/−^ BALB/c mice. The number of clones (n) is indicated. Sequences were obtained from independent clones from five tumors in different animals. (A) The number of G-to-A mutations on the proviral plus strand by Sanger sequencing. (B) The number of C-to-T mutations on the proviral plus strand by Sanger sequencing. (C to F) The number of C mutations within different motifs on either proviral strand is presently graphically for each clone. (C) The number of mutations/clone in the WRC motif typical of AID mutation hot spots. (D) The number of mutations/clone in the SYC motif. (E) The number of mutations/clone in the TYC motif typical of mA3 mutation hot spots. (F) The number of mutations/clone in the ATC motif. Statistical significance of results of nonparametric Mann-Whitney tests is indicated on the scatter plots. The mutant C residue in each motif is underlined.

To directly analyze differences in distribution of specific MMTV proviral mutations in the presence and absence of AID, we compared the numbers of mutations/clone in MMTV-WT proviruses from mammary tumors in BALB/c mice versus *Aicda*^−/−^ mice (Fig. 5). None were significantly different. In contrast, the number of C-to-T mutations as well as WRC and TYC motif mutations within MMTV-SD proviruses (MMTV-SD recombinants and MMTV-SD non-recombinants combined) significantly declined in *Aicda*^−/−^ mice compared to wild-type BALB/c mice (Fig. 5B, C, and E), whereas those in SYC motifs increased in AID-knockout mice (Fig. 5D). Only mutations in the ATC motif showed a marginal (but insignificant) decline when comparing the number of proviral MMTV-SD mutations/clone between BALB/c and *Aicda*^−/−^ mice (*P* = 0.08) (Fig. 5F). These results are consistent with the interpretation that the absence of Rem expression leads to increased mutations by AID, similar to HIV-1 proviral hypermutation by A3G in the absence of Vif (16, 51).

### AID, but not mA3, proteasomal degradation in the presence of rem.

**FIG 6:**
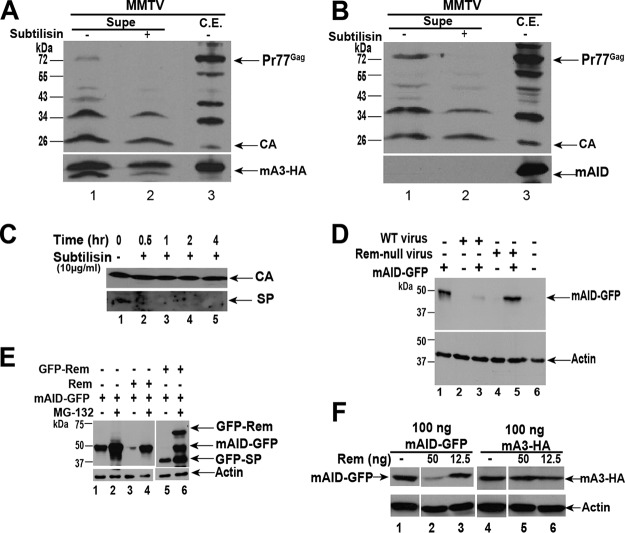
Rem antagonizes AID. (A) mA3-HA is detectable in MMTV virions. Cells (293T) were cotransfected with MMTV-expressing and mA3-HA-expressing plasmids. Western blots for both cell extracts (C.E.) and the 100-fold concentrated cell supernatant (with or without subtilisin) are shown. Blots were incubated with either MMTV CA-specific antibody (upper panel) or HA-specific antibody (lower panel). (B) AID is not detectable in MMTV virions. Cells (293T) were cotransfected with MMTV-expressing and AID-expressing plasmids. Western blots for both cell extracts (C.E.) and the 100-fold concentrated cell supernatant (with or without subtilisin) are shown. Blots were incubated with either MMTV CA-specific antibody (upper panel) or AID-specific antibody (lower panel). (C) SP is not packaged into wild-type virions. Concentrated TBLV-WT virions were treated for the indicated times with subtilisin to remove cellular proteins external to the virion envelope. Western blots of supernatants were incubated with MMTV CA-specific antibodies (upper panel) or SP-specific antibodies (lower panel). (D) Cotransfection of Rem-expressing provirus leads to reduced mAID-GFP levels, whereas cotransfection of a Rem-null provirus does not. Either TBLV-WT or TBLV-SD expression plasmid was transfected into Jurkat cells in the presence or absence of murine AID tagged with GFP. Western blots were performed with GFP-specific antibody (upper panel) or actin-specific antibody (lower panel). (E) Rem expression leads to decreased mAID-GFP levels and is dependent on the proteasome. Cells (293 line) were cotransfected with expression plasmids for mAID-GFP and either GFP-tagged or untagged Rem. Samples in even-numbered lanes were prepared from cells treated with the proteasomal inhibitor MG-132. Western blots of cell extracts were incubated with GFP-specific or actin-specific antibody (upper and lower panels, respectively). (F) Rem expression does not affect mA3-HA levels. Cells (293 line) were transfected with the indicated amount of mAID-GFP expression vector or mA3-HA in the presence or absence of the indicated amount of untagged Rem expression plasmid. The blot shown at upper left was incubated with GFP-specific antibody, and that shown at upper right was incubated with HA-specific antibody. Actin-specific antibody (lower panels) was used to verify protein loading.

APOBEC3 enzymes that are incorporated into HIV-1 virions in the absence of Vif activity deaminate cytidines on negative-strand DNA during reverse transcription in the target cell (12). Vif expression targets APOBEC3 for proteasomal degradation (53), leading to decreased deaminase packaging and proviral mutagenesis. Since murine AID belongs to the Apobec family (12), we determined whether AID is packaged into MMTV virions. As a control for deaminase packaging, 293T cells were transiently transfected with expression plasmids for cytomegalovirus (CMV) promoter-driven MMTV-WT as well as mA3 tagged with hemagglutinin (mA3-HA). Previous experiments have shown that the mA3 deaminase is packaged in MMTV particles isolated from virus-containing milk (22). Virus released from 293T cells was concentrated, and Western blotting was performed on concentrated cell supernatants after treatment with subtilisin to remove cellular proteins on the surface of virions. MMTV Gag-specific antibody detected capsid (CA) proteins both in the cell extracts and in the concentrated supernatants. As expected, we also observed mA3 in cell extracts and in supernatants (Fig. 6A). The presence of mA3 remained detectable after subtilisin treatment, confirming that mA3 is present within viral cores (22).

Similar experiments were performed to determine whether AID was packaged in MMTV particles by cotransfection of CMV–MMTV-WT and murine AID (mAID) into 293T cells. Enrichment of the cleaved Gag protein was easily observed in cell supernatants containing virion particles compared to extracts (Fig. 6B). Under these conditions, Western blotting with AID-specific antibody easily detected mAID in cell extracts, but not in cell supernatants (Fig. 6B). To confirm the effectiveness of the subtilisin, Rev-like SP protein also was not detectable in viral cores after treatment (Fig. 6C). These experiments suggest that AID induces MMTV hypermutation without virion incorporation.

Since HIV-1 Vif expression induces proteasomal degradation of specific APOBEC3 enzymes (53), we tested whether Rem expression from the wild-type provirus affects AID levels. Human Jurkat T cells were transfected with a plasmid expressing murine AID with a C-terminal green fluorescent protein (GFP) (mAID-GFP) in the presence or absence of the wild-type provirus (Fig. 6D). Western blotting of transfected cell extracts and incubation with GFP-specific antibody showed mAID-GFP levels that were greatly diminished by the presence of the Rem-expressing genome (compare lanes 1 and 3). In contrast, cotransfection with the Rem-null provirus did not affect AID levels (lane 5).

To confirm that Rem expression was responsible for decreased AID levels, we cotransfected HEK293 cells with N-terminally GFP-tagged Rem or untagged Rem with mAID-GFP. The presence of either form of Rem protein decreased detectable AID levels (Fig. 6E; compare lane 1 with lane 3 or lane 5); AID levels were rescued in the presence of the proteasomal inhibitor MG-132 (Fig. 6E, even-numbered lanes). As previously reported, the GFP-Rem precursor was stabilized more than the cleaved GFP-SP product due to precursor susceptibility to endoplasmic reticulum-associated degradation (ERAD) (compare lanes 5 and 6) (6). These results are consistent with Vif-like activity of Rem. Since we also detected mutations in mA3 motifs preferentially in proviruses from Rem-null virus-induced tumors, we transfected HEK293 cells with Rem expression vectors in the presence of plasmids expressing mAID-GFP or mA3 C-terminally tagged with HA (mA3-HA). As expected, higher Rem levels led to a greater reduction of mAID levels (Fig. 6F, compare lanes 1, 2, and 3). In contrast, the same Rem concentrations had no effect on mA3 expression (Fig. 6F, compare lanes 4, 5, and 6). Thus, our data indicate that mAID is targeted for proteasomal degradation in the presence of Rem, whereas mA3 is not.

## DISCUSSION

Previous data from studies of mA3-insufficient mice indicated that MMTV replication and tumorigenesis are inhibited by members of the Apobec family of cytidine deaminases (12). No MMTV-specified inhibitors of these enzymes have been reported, including in a recent report that concluded on the basis of transfection experiments in 293T cells that MMTV does not encode an mA3 inhibitor (54). Our experiments are consistent with the need for Rem C-terminal sequences to antagonize the mutagenic effects of the Apobec cytidine deaminase AID during MMTV replication in lymphocytes.

Multiple results support this conclusion. (i) Tumors induced by wild-type MMTV had low, but reproducibly higher, proviral loads than tumors induced by viral strains that lack Rem expression (MMTV-SD). We found that the difference in proviral loads was abolished in tumors induced by MMTV in *Aicda*^−/−^ mice, suggesting that AID is a restriction factor for MMTV (Fig. 1 and Fig. 4). (ii) Loss of Rem expression led to an increase in G-to-A mutations as well as in other transition mutations on the proviral plus strand (Table 1 and Table 2). (iii) Mutation frequency and numbers of mutations per proviral clone in the WRC motif (typical of AID) were elevated in tumors induced by MMTV-SD proviruses compared to tumors induced by MMTV-WT proviruses. This mutation pattern was observed in proviruses obtained from wild-type BALB/c mouse tumors, but not *Aicda*^−/−^ BALB/c mouse tumors (Table 1 and Table 3) (Fig. 5). (iv) Tumors induced by a second Sag-independent MMTV strain, TBLV-WT, also showed an increase in proviral load compared to tumors induced by the Rem-defective virus (TBLV-SD) (Fig. 3). Increased mutations in WRC motifs were observed in TBLV-SD proviruses compared to TBLV-WT proviruses using Sanger sequencing of cloned proviruses (Fig. 3) (Table 2). Although MMTV-SD is defective for production of both *sag* mRNA from the intragenic *env* promoter and *rem* mRNA (Fig. 1C) (9), TBLV is a Sag-independent virus (41) and does not replicate in the mammary gland. Thus, loss of Rem expression, and not loss of Sag expression, is responsible for inhibition of MMTV replication by cytidine deamination in hematopoietic cells (Fig. 3) (Table 2). (v) Rem coexpression led to proteasomal degradation of AID, but not mA3 (Fig. 6). Our results suggest that Rem specifies a Vif-like factor (12) that antagonizes the AID restriction factor in hematopoietic cells prior to mammary gland transmission (3).

We observed increased mutations within proviruses obtained from MMTV-SD-induced mammary tumors or from TBLV-SD-induced lymphomas within TYC motifs (typical of mA3) as well as WRC motifs (typical of AID). Mutations in both motifs greatly decreased in tumor-derived MMTV proviruses from AID-insufficient mice relative to AID-expressing mice (compare Table 1 to Table 3 and Fig. 2 to Fig. 4). Since Rem coexpression did not affect mA3 levels, one interpretation is that AID causes both WRC-motif mutations and TYC-motif mutations. However, the number of WRC-motif mutations/clone increased in TBLV-SD recombinants from BALB/c mice, whereas the number of TYC mutations/clone decreased (compare Fig. 3E and G), suggesting that these mutations may be due to the activity of different enzymes. Furthermore, the number of TYC mutations/clone increased in TBLV-SD non-recombinants relative to TBLV-WT (Fig. 3G), but the same was not true for the WRC motif (Fig. 3E). Also, AID-mediated TYC-motif mutations have not been described in immunoglobulin genes (55), although the mechanism of AID-induced proviral mutations may be different.

Previous studies have suggested that sequence changes at the SYC motif represent AID-induced “cold spot” mutations (38, 55). Since *Aicda*^−/−^ mice showed an increase in the level of SYC-motif mutations in proviruses from MMTV-SD-induced tumors (Table 3) (Fig. 4), our results imply that these mutations are due to the activity of an unidentified cytidine deaminase. In addition, proviral mutations in the ATC motif did not segregate with WRC or TYC-motif mutations either in MMTV-SD-induced mammary tumors from *Aicda*^−/−^ mice (Fig. 4) or in TBLV-SD-induced T-cell tumors from wild-type BALB/c mice (Fig. 3). Possible explanations for our data include: (i) decreased AID levels directly or indirectly affect the activity of other Apobec family members, such as mA3, or (ii) the *Aicda* gene knockout mutation affects the types of cells infected by MMTV. Infection of additional Apobec-knockout mouse strains should address this issue.

**FIG 7:**
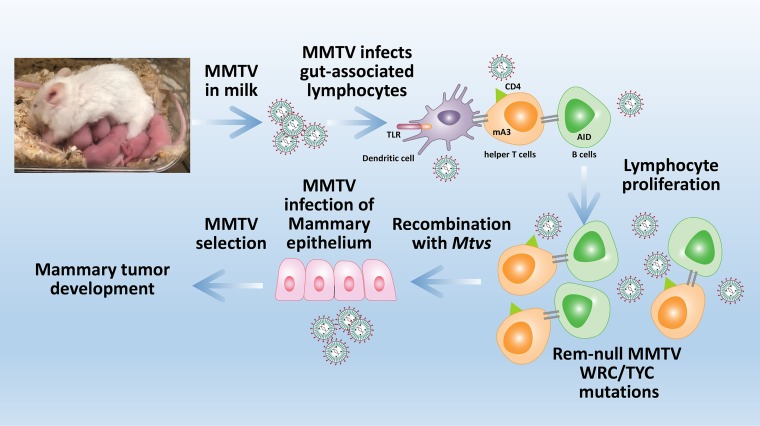
Model for Apobec-mediated mutations during MMTV replication *in vivo*. MMTV in maternal milk infects dendritic cells (DCs) in the gut of newborn animals. Virus is transmitted from DCs to T cells, which then likely transmit MMTV to B cells. MMTV-encoded superantigen (Sag) expression on mature B cells (gray lines) is required for efficient MMTV transmission from infected B and T lymphocytes to mammary cells during puberty. Gray lines also depict proteins mediating B-cell and T-cell interactions. Repeated proviral insertions into mammary cells are required to get MMTV insertional activation of proto-oncogenes and mammary tumors. Apobec-mediated proviral mutations, including those induced by AID, occur in the absence of the antagonist Rem during both MMTV-SD replication and TBLV-SD replication in lymphocytes. Recombination within lymphocytes of endogenous *Mtv*s with SD mutants allows recovery of Rem expression. TLR, Toll-like receptor.

One striking observation is the enrichment of sequence alterations within Apobec-specific motifs in proviruses that had repaired the SD mutation. SD-site reversion occurred at all 6 modified bases, probably by recombination with one of the complete endogenous proviruses in BALB/c mice as observed previously for other MMTV-induced tumors (9, 56, 57). The high percentage of recombinants indicates strong selection for the restoration of splicing at the *env* gene SD site, which results in production of either *rem* mRNA from the LTR promoter or *sag* mRNA from the intragenic *env* promoter (9, 44) (Fig. 1C). We believe that SD-site reversion does not select for wild-type envelope protein production since the SD mutation produces a single valine-to-leucine change. Moreover, proviral loads differed only between MMTV-induced-SD and WT-induced tumors in BALB/c mice and not between those in *Aicda*^−/−^ mice (Fig. 2 and Fig. 4), indicating the absence of an Env-specific replication effect *in vivo* when the selective effect of AID expression was removed. SD site reversion is not due to *sag* expression in T-cell tumors since TBLV does not encode a functional Sag protein (41). Interestingly, the number of recombinants regenerating the SD site correlated with the numbers of TBLV and MMTV proviral mutations in either WRC or TYC motifs in tumors in AID-sufficient mice (Fig. 3I and J), suggesting that recombination and mutation may occur in the same cells. These mutations likely occur in lymphocytes prior to mammary gland transmission since (i) Sag-mediated T-cell deletion is delayed in MMTV-SD-infected BALB/c mice, as we previously showed (9); (ii) endogenous *Mtv*s capable of recombination with exogenous MMTV are expressed specifically in lymphocytes and not in mammary gland cells (58); and (iii) TBLV replicates at high levels in T cells and does not induce mammary cancer (47, 49). We speculate that virus replicating in B and T cells is mutated by AID and other Apobec enzymes prior to mutant RNA packaging with endogenous *Mtv* RNA and subsequent recombination during the next round of reverse transcription. Therefore, the appearance of SD site recombinants in both MMTV-induced and TBLV-induced tumors argues that Rem production provides a selective advantage for virus propagation of both Sag-dependent and Sag-independent viruses. The appearance of larger numbers of recombinant MMTV proviruses without stop codons in the envelope gene compared to TBLV recombinant proviruses argues that heavy selection of MMTV proviruses occurred during viral transmission to mammary tissue (see model in Fig. 7). MMTV replication-independent activation of B cells has been reported (59), and TBLV may also activate and infect B cells without superantigen. Additional experiments analyzing proviral mutations and recombination with endogenous *Mtv*s in different lymphocyte subsets are needed.

Results presented here indicated that AID, but not mA3, is degraded after coexpression with GFP-tagged or untagged Rem (Fig. 6). AID degradation by Rem was rescued by the proteasomal inhibitor MG-132, a result reminiscent of HIV-1 Vif activity on human APOBEC3G (hA3G) (16–19). Vif has been shown to act as an adapter between hA3G and a Cullin5 E3 ligase complex, resulting in hA3G ubiquitylation and proteasomal degradation (52, 60). These results strongly suggest that Rem is a Vif-like factor that antagonizes the restriction factor AID. Unlike Vif, Rem is synthesized in association with ER membranes (6, 8, 61), where this precursor is cleaved by signal peptidase to produce SP and Rem-CT (Fig. 1) or retrotranslocated to the cytosol by the p97 ATPase for ERAD (6, 8). Further work will be necessary to determine whether cleaved Rem-CT or uncleaved Rem is required for AID proteasomal degradation, the nature of the E3 ligase involved in mAID degradation, the cellular location of mAID during Rem-mediated degradation, and whether Rem and mAID have a direct interaction. In addition, the lack of mAID packaging into MMTV particles suggests that this cytidine deaminase functions differently on the MMTV genome from human APOBEC enzymes on the HIV genome (62, 63). One possibility is that mAID acts on the proviral genome during preintegration complex transit into the nucleus (64).

In summary, our data are consistent with the conclusion that Rem is the first retrovirus-encoded protein antagonist of a deamination-dependent AID activity to be identified. AID is believed to be the primordial member of the Apobec family (12), which likely evolved to antagonize the mutagenic activity of retrotransposons (65), and yet has also been shown to be important for restricting replication of herpesviruses in B cells (66). The original role of AID in retrotransposon control may have later diversified to regulate various aspects of adaptive immunity to infectious agents, including antibody hypermutation and class switch recombination (28, 67, 68). Since AID overexpression has been associated with multiple human cancers (29), particularly B-cell lymphomas (69, 70), elucidation of the Rem-mediated mechanism targeting AID for degradation may be useful for treatment of both human tumors (29, 69) and infectious diseases.

## MATERIALS AND METHODS

### Cell lines and transfections.

The conditions used for growth and transfection of rat XC fibroblasts, human Jurkat T-cell lymphoma, human embryonic kidney (HEK293 and 293T), and mouse mammary HC11 cells have been described previously (71). Transfected XC cells were cultured with dexamethasone to induce the hormone-responsive MMTV promoter (2).

### Mouse experiments.

Female BALB/cJ mice were injected intraperitoneally with 2 × 10^7^ Jurkat T cells producing TBLV-WT or TBLV-SD or with 2 × 10^7^ hormone-induced XC cells producing MMTV-WT or MMTV-SD according to our previously established method (41, 49). *Aicda*^−/−^ mice on the BALB/cJ background were derived by 10 backcross generations with *Aicda*^−/−^ mice on the C57BL/6 background obtained from T. Honjo, Kyoto University (28) (kindly provided by M. Nussenzweig, Rockefeller University). Males were selected on the basis of 80 markers that distinguished between BALB/c and C57BL/6 backgrounds. All mice were confirmed to have the correct genotype by PCR-based typing of tail DNA as previously described (72). Injected mice were monitored for the appearance of T-cell lymphomas or mammary carcinomas, and the resulting tumors were frozen at –70°C. Tumors were extracted for high-molecular-mass DNA as previously described (73). All animal experiments were approved by the University of Texas at Austin (UT Austin) IACUC.

### Constructs and reporter assays.

Infectious clone pHYB-TBLV-Hyg has been previously shown to induce T-cell lymphomas (41). The 1.3-kb BamHI *env* fragment from pHYB-MTV-SD containing a mutation in the SD site (9) was used to substitute for the same fragment in pHYB-TBLV-Hyg to construct pHYB-TBLV-SD-Hyg. The wild-type *env* sequence 5′-CAA GGG GTG AGT-3′ (Gln-Gly-Val-Ser) was changed to 5′-CAG GGA CTC TCA-3′ (Gln-Gly-Leu-Ser) in the SD sequence (9). The GFP-Rem-T7 construct has an N-terminal enhanced GFP (EGFP) tag and a C-terminal glycine linker followed by the T7 tag. The constructs were confirmed by restriction enzyme digestion and sequencing. The C-terminally tagged murine AID expression plasmid (AID-GFP) (74) was obtained from Fred Alt. The pcDNA3-AID plasmid was cloned from pMX-AID, which was obtained from Kevin McBride (75). CMV-MMTV and pcDNA3-mA3-HA plasmids were kindly provided by Farah Mustafa (76) and Nathaniel Landau (77), respectively.

The GFP-Rem expression plasmid and pHM*Rluc* Rem-responsive reporter constructs have been described previously (1). *Renilla* reporter activity was determined from triplicate transfections, and the data were normalized to Rem-nonresponsive firefly luciferase levels (pGL3-Control; Promega) to control for differences in transfection efficiency. Activity was assessed per 100 μg of protein using a Dual Luciferase assay kit (Promega). The means of results of triplicate transfections ± standard deviations are shown.

### Virus packaging experiments.

MMTV was produced in 293T cells by transfection of the CMV-MMTV plasmid (76). Incorporation of AID and mA3 in MMTV particles was tested by cotransfecting CMV-MMTV (1 μg) with pcDNA3-AID (untagged) or pcDNA3-mA3-HA (2 μg). Large amounts of AID expression plasmid were transfected to overcome Rem-mediated proteasomal degradation and to maximize detectable levels of packaging. After centrifugation at 4,000 × *g* to remove any cell contaminants, supernatants were passed through a 0.45-μm-pore-size filter and incubated overnight with one-third volume of RetroX concentrator (TaKaRa) at 4°C. Virus precipitates were pelleted by centrifugation at 1,500 × *g* for 45 min at 4°C and resuspended in phosphate-buffered saline (PBS). For protease treatment, half of the PBS resuspensions were incubated with subtilisin (Roche) at a final concentration of 10 μg/ml for 1 h at 37°C. Subsequently, the samples were analyzed by Western blotting with antibodies specific for CA, HA, or AID.

### Western blotting.

Western blots were performed essentially as described previously (7). Polyclonal MMTV SP-specific antibodies were prepared in rabbits as described previously (6). Rem-CT-specific antibodies were prepared by intraperitoneal injections of the peptide TVDNNKPGGKGDKRRM after conjugation to carrier protein and multiple injections into rabbits (New England Peptide). Antibodies to AID and GAPDH (glyceraldehyde-3-phosphate dehydrogenase) were obtained from Cell Signaling. Antibodies specific for HA, GFP, T7 tag, and actin were purchased from BioLegend, Clontech, Novagen, and Calbiochem, respectively. MMTV Gag-specific monoclonal antibodies were kindly provided by Tanya Golovkina (University of Chicago) (78). Secondary antibodies were obtained from Jackson Immunoresearch.

### RT-PCR.

RNA was extracted from Jurkat cells stably producing TBLV-WT or TBLV-SD and XC cells stably expressing MMTV-WT or MMTV-SD by the guanidine isothiocyanate method, treated with DNase, and used for reverse transcription with poly(dT)_17_ in the presence of RNaseOut (Invitrogen) as described previously (7). The cDNA was used with primers C3H 230+ (5′-GTG AAT TCC ATC ACA AGA GCG GAA CGG AC-3′) and C3H LTR420− (5′-GAT TCA TTT CTT AAC ATA GTA AC-3′) in reaction mixtures with JumpStart REDTaq reaction mix (Sigma-Aldrich). The PCR parameters were as follows: 94°C for 2 min and then 30 cycles of 94°C for 20 s, 55°C for 30 s, and 68°C for 6 min and an 8-min extension at 68°C. Reactions were analyzed on 1% agarose gels and stained with ethidium bromide prior to visualization using an AlphaImager (Alpha Innotech).

### PCR and high-throughput sequencing.

DNA extracted from tumors induced by TBLV-WT and TBLV-SD (3 tumors each) was used for PCR with primers *env*7254(+) (5′-ATC GCC TTT AAG AAG GAC GCC TTC T-3′) and LTR9604(−) (5′-GGA AAC CAC TTG TCT CAC ATC-3′) for the envelope region, whereas the primers used for the polymerase region were *pol*4235(+) (5′-GAA GAG AGC AAT AGC CCT TG-3′) and *pol*5835(−) (5′-GAT GAT GTA GTG CGT GGC-3′). For DNA extracted from tumors induced by MMTV-WT and MMTV-SD, C3H LTR420− (5′-GAT TCA TTT CTT AAC ATA GTA AC-3′) was used as the reverse primer for *env* gene amplification. The primers for *GAPDH* have been described previously (1). *c-Myc* was amplified using primers *c-Myc*(+) (5′-ATG CCC CTC AAC GTG AAC TT-3′) and *c-Myc*(−) (5′-AGG AGG TCC ATC CAA CCT CT-3′). Reactions were performed with JumpStart RED Accutaq LA polymerase (Sigma-Aldrich) in a reaction mixture consisting of the supplied buffer, 500 ng of tumor DNA, 50 pmol of each primer, and a 0.5 mM concentration of each deoxynucleoside triphosphate in 20 μl. PCR parameters were: 94°C for 1 min followed by 10 cycles at 94°C for 10 s, 53°C for 30 s, and 68°C for 2 min and then by 25 cycles of 95°C for 15 s, 50°C for 30 s, and 68°C for 2 min and then a final incubation at 68°C for 7 min. Five independent reaction mixtures corresponding to each tumor DNA were pooled for each primer set (except for *GAPDH*) and used for Illumina sequencing at the UT Austin Genomic Sequencing and Analysis Facility. These results were confirmed by independent cloning of PCR fragments encompassing the SD site and the *env* gene followed by Sanger sequencing. Sequencing also enabled identification of proviruses containing SD recombinants. Semi-quantitative PCR was performed with *Mtvr2* as the single-copy gene standard using primers *Mtvr2*(+) (5′-TCT GGG ATC CGC TTC CTC AT-3′) and *Mtvr2*(−) (5′-CCA GTC CTT GGC CCT CAT TTA-3′). MMTV primers *pol*4235(+) and *pol*5835(−) in the viral polymerase gene were used to measure proviral sequences.

### Sequencing motifs and identification software.

Mutations in the WRC and SYC sequence motifs have been associated with “hot spots” and “cold spots”, respectively, for AID-mediated hypermutations (36–38). Mutations in the TYC sequence motif have been linked to mA3 activity (23, 39); mutations in the ATC sequence motif have also been associated with mA3 activity on synthetic templates (40). The software TransitionFinder was developed to identify sites of G-to-A and C-to-T mismatches between a given reference FASTA sequence and a user-generated set of FASTA sequences (test sequences). The script is publicly available at https://github.com/haridh/Dudley_Lab_Collab.

### Statistical analysis.

All experiments were performed at least twice with similar results. Statistical differences between tumor development induced by the wild-type and mutant viruses and Kaplan-Meier curves were calculated using SPSS software and the log rank (Mantel-Cox) test with the consulting services of the Department of Statistics and Data Sciences at The University of Texas at Austin. Two-tailed *t* tests were used for pairwise comparisons. Differences in distributions of numbers of mutations/clone for scatter plots were assessed by nonparametric Mann-Whitney tests. The wide range of variations of mutations/clone prevented statistical comparison of means or medians. Correlation analysis of nonparametric data was performed by calculating Spearman’s correlation coefficients. Statistical significances of differences are indicated, and a value of <0.05 was considered to be significant. Values for numbers of samples analyzed (*n*) are indicated within individual figures.

For high-throughput Illumina data, the aligned paired-end sequences from the *env*-3′ LTR and *pol* PCR products were aligned to the reference HYB-TBLV molecular clone using BWA-MEM (79). Likely PCR or optical duplicates were marked using Picard MarkDuplicates (https://broadinstitute.github.io/picard/) and removed using SAMtools (80). The counts of reads aligning to each base in the reference were determined using bam-readcount (https://github.com/genome/bam-readcount). All positions lacking reads were filtered out and were not included in further analysis. Read counts were visualized using ggplot2 (81) and then modeled with respect to dichotomized alternative base frequency using a 3% threshold for PCR error as a function of sample group (either TBLV-WT or TBLV-SD). Modeling was performed with a mixed-effect logistic regression model for each combination of reference base (A, C, G, or T) and alternative base. A similar analysis was performed for the Mus musculus *Gapdh* sequences (NM_008084.2) obtained by PCR from TBLV-WT-induced or TBLV-SD-induced tumors.

### Data availability.

We have submitted the high-throughput data to GEO (GSE134189). We also submitted to GenBank the primary sequence data for the TBLV molecular clone used to produce tumors in mice (GenBank accession no. MN126120). The Sanger sequence data are available by request.

## References

[1] Mertz JA, Simper MS, Lozano MM, Payne SM, Dudley JP 2005 Mouse mammary tumor virus encodes a self-regulatory RNA export protein and is a complex retrovirus. J Virol 79:14737–14747. doi:10.1128/JVI.79.23.14737-14747.2005. 16282474 PMC1287593

[2] Dudley JP, Golovkina TV, Ross SR 2016 Lessons learned from mouse mammary tumor virus in animal models. ILAR J 57:12–23. doi:10.1093/ilar/ilv044. 27034391 PMC5007637

[3] Golovkina TV, Dudley JP, Jaffe AB, Ross SR 1995 Mouse mammary tumor viruses with functional superantigen genes are selected during in vivo infection. Proc Natl Acad Sci U S A 92:4828–4832. doi:10.1073/pnas.92.11.4828. 7761408 PMC41800

[4] Bergman AC, Björnberg O, Nord J, Nyman PO, Rosengren AM 1994 The protein p30, encoded at the gag-pro junction of mouse mammary tumor virus, is a dUTPase fused with a nucleocapsid protein. Virology 204:420–424. doi:10.1006/viro.1994.1547. 8091672

[5] Indik S, Günzburg WH, Salmons B, Rouault F 2005 A novel, mouse mammary tumor virus encoded protein with Rev-like properties. Virology 337:1–6. doi:10.1016/j.virol.2005.03.040. 15914215

[6] Byun H, Halani N, Mertz JA, Ali AF, Lozano MM, Dudley JP 2010 Retroviral Rem protein requires processing by signal peptidase and retrotranslocation for nuclear function. Proc Natl Acad Sci U S A 107:12287–12292. doi:10.1073/pnas.1004303107. 20566871 PMC2901445

[7] Mertz JA, Lozano MM, Dudley JP 2009 Rev and Rex proteins of human complex retroviruses function with the MMTV Rem-responsive element. Retrovirology 6:10. doi:10.1186/1742-4690-6-10. 19192308 PMC2661877

[8] Byun H, Das P, Yu H, Aleman A, Lozano MM, Matouschek A, Dudley JP 2017 Mouse mammary tumor virus signal peptide uses a novel p97-dependent and Derlin-independent retrotranslocation mechanism to escape proteasomal degradation. mBio 8:e00328-17. doi:10.1128/mBio.00328-17. 28351922 PMC5371415

[9] Mustafa F, Lozano M, Dudley JP 2000 C3H mouse mammary tumor virus superantigen function requires a splice donor site in the envelope gene. J Virol 74:9431–9440. doi:10.1128/jvi.74.20.9431-9440.2000. 11000212 PMC112372

[10] Beutner U, Kraus E, Kitamura D, Rajewsky K, Huber BT 1994 B cells are essential for murine mammary tumor virus transmission, but not for presentation of endogenous superantigens. J Exp Med 179:1457–1466. doi:10.1084/jem.179.5.1457. 8163931 PMC2191484

[11] Golovkina TV, Chervonsky A, Dudley JP, Ross SR 1992 Transgenic mouse mammary tumor virus superantigen expression prevents viral infection. Cell 69:637–645. doi:10.1016/0092-8674(92)90227-4. 1316806

[12] Harris RS, Dudley JP 2015 APOBECs and virus restriction. Virology 479–480:131–145. doi:10.1016/j.virol.2015.03.012. PMC4424171 25818029

[13] Stavrou S, Nitta T, Kotla S, Ha D, Nagashima K, Rein AR, Fan H, Ross SR 2013 Murine leukemia virus glycosylated Gag blocks apolipoprotein B editing complex 3 and cytosolic sensor access to the reverse transcription complex. Proc Natl Acad Sci U S A 110:9078–9083. doi:10.1073/pnas.1217399110. 23671100 PMC3670389

[14] Rosales Gerpe MC, Renner TM, Bélanger K, Lam C, Aydin H, Langlois M-A 2015 N-linked glycosylation protects gammaretroviruses against deamination by APOBEC3 proteins. J Virol 89:2342–2357. doi:10.1128/JVI.03330-14. 25505062 PMC4338886

[15] Kolokithas A, Rosenke K, Malik F, Hendrick D, Swanson L, Santiago ML, Portis JL, Hasenkrug KJ, Evans LH 2010 The glycosylated Gag protein of a murine leukemia virus inhibits the antiretroviral function of APOBEC3. J Virol 84:10933–10936. doi:10.1128/JVI.01023-10. 20702647 PMC2950561

[16] Sheehy AM, Gaddis NC, Malim MH 2003 The antiretroviral enzyme APOBEC3G is degraded by the proteasome in response to HIV-1 Vif. Nat Med 9:1404–1407. doi:10.1038/nm945. 14528300

[17] Conticello SG, Harris RS, Neuberger MS 2003 The Vif protein of HIV triggers degradation of the human antiretroviral DNA deaminase APOBEC3G. Curr Biol 13:2009–2013. doi:10.1016/j.cub.2003.10.034. 14614829

[18] Stopak K, de Noronha C, Yonemoto W, Greene WC 2003 HIV-1 Vif blocks the antiviral activity of APOBEC3G by impairing both its translation and intracellular stability. Mol Cell 12:591–601. doi:10.1016/S1097-2765(03)00353-8. 14527406

[19] Marin M, Rose KM, Kozak SL, Kabat D 2003 HIV-1 Vif protein binds the editing enzyme APOBEC3G and induces its degradation. Nat Med 9:1398–1403. doi:10.1038/nm946. 14528301

[20] Iwatani Y, Chan DSB, Wang F, Maynard KS, Sugiura W, Gronenborn AM, Rouzina I, Williams MC, Musier-Forsyth K, Levin JG 2007 Deaminase-independent inhibition of HIV-1 reverse transcription by APOBEC3G. Nucleic Acids Res 35:7096–7108. doi:10.1093/nar/gkm750. 17942420 PMC2175344

[21] Mangeat B, Turelli P, Caron G, Friedli M, Perrin L, Trono D 2003 Broad antiretroviral defence by human APOBEC3G through lethal editing of nascent reverse transcripts. Nature 424:99–103. doi:10.1038/nature01709. 12808466

[22] Okeoma CM, Lovsin N, Peterlin BM, Ross SR 2007 APOBEC3 inhibits mouse mammary tumour virus replication in vivo. Nature 445:927–930. doi:10.1038/nature05540. 17259974

[23] Langlois M-A, Kemmerich K, Rada C, Neuberger MS 2009 The AKV murine leukemia virus is restricted and hypermutated by mouse APOBECIII. J Virol 83:11550–11559. doi:10.1128/JVI.01430-09. 19726503 PMC2772689

[24] Boi S, Kolokithas A, Shepard J, Linwood R, Rosenke K, Van Dis E, Malik F, Evans LH 2014 Incorporation of mouse APOBEC3 into murine leukemia virus virions decreases the activity and fidelity of reverse transcriptase. J Virol 88:7659–7662. doi:10.1128/JVI.00967-14. 24719421 PMC4054435

[25] Boi S, Ferrell ME, Zhao M, Hasenkrug KJ, Evans LH 2018 Mouse *Apobec3* expression in NIH 3T3 cells mediates hypermutation of AKV murine leukemia virus. Virology 518:377–384. doi:10.1016/j.virol.2018.03.014. 29605684 PMC5918422

[26] Bednarski JJ, Sleckman BP 2019 At the intersection of DNA damage and immune responses. Nat Rev Immunol 19:231–242. doi:10.1038/s41577-019-0135-6. 30778174 PMC6438741

[27] Sherman MH, Kuraishy AI, Deshpande C, Hong JS, Cacalano NA, Gatti RA, Manis JP, Damore MA, Pellegrini M, Teitell MA 2010 AID-induced genotoxic stress promotes B cell differentiation in the germinal center via ATM and LKB1 signaling. Mol Cell 39:873–885. doi:10.1016/j.molcel.2010.08.019. 20864035 PMC2945612

[28] Muramatsu M, Kinoshita K, Fagarasan S, Yamada S, Shinkai Y, Honjo T 2000 Class switch recombination and hypermutation require activation-induced cytidine deaminase (AID), a potential RNA editing enzyme. Cell 102:553–563. doi:10.1016/S0092-8674(00)00078-7. 11007474

[29] Honjo T, Kobayashi M, Begum N, Kotani A, Sabouri S, Nagaoka H 2012 The AID dilemma: infection, or cancer? Adv Cancer Res 113:1–44. doi:10.1016/B978-0-12-394280-7.00001-4. 22429851

[30] Held W, Shakhov AN, Izui S, Waanders GA, Scarpellino L, MacDonald HR, Acha-Orbea H 1993 Superantigen-reactive CD4+ T cells are required to stimulate B cells after infection with mouse mammary tumor virus. J Exp Med 177:359–366. doi:10.1084/jem.177.2.359. 8093892 PMC2190911

[31] Bishop KN, Holmes RK, Sheehy AM, Davidson NO, Cho S-J, Malim MH 2004 Cytidine deamination of retroviral DNA by diverse APOBEC proteins. Curr Biol 14:1392–1396. doi:10.1016/j.cub.2004.06.057. 15296758

[32] Jern P, Stoye JP, Coffin JM 2007 Role of APOBEC3 in genetic diversity among endogenous murine leukemia viruses. PLoS Genet 3:2014–2022. doi:10.1371/journal.pgen.0030183. 17967065 PMC2041998

[33] Wu X, Feng J, Komori A, Kim EC, Zan H, Casali P 2003 Immunoglobulin somatic hypermutation: double-strand DNA breaks, AID and error-prone DNA repair. J Clin Immunol 23:235–246. doi:10.1023/A:1024571714867. 12959216 PMC4624321

[34] Zan H, Wu X, Komori A, Holloman WK, Casali P 2003 AID-dependent generation of resected double-strand DNA breaks and recruitment of Rad52/51 in somatic hypermutation. Immunity 18:727–738. doi:10.1016/S1074-7613(03)00151-1. 12818155 PMC4625537

[35] Muramatsu M, Sankaranand VS, Anant S, Sugai M, Kinoshita K, Davidson NO, Honjo T 1999 Specific expression of activation-induced cytidine deaminase (AID), a novel member of the RNA-editing deaminase family in germinal center B cells. J Biol Chem 274:18470–18476. doi:10.1074/jbc.274.26.18470. 10373455

[36] Yu K, Huang F-T, Lieber MR 2004 DNA substrate length and surrounding sequence affect the activation-induced deaminase activity at cytidine. J Biol Chem 279:6496–6500. doi:10.1074/jbc.M311616200. 14645244

[37] Rogozin IB, Diaz M 2004 Cutting edge: DGYW/WRCH is a better predictor of mutability at G:C bases in Ig hypermutation than the widely accepted RGYW/WRCY motif and probably reflects a two-step activation-induced cytidine deaminase-triggered process. J Immunol 172:3382–3384. doi:10.4049/jimmunol.172.6.3382. 15004135

[38] Wei L, Chahwan R, Wang S, Wang X, Pham PT, Goodman MF, Bergman A, Scharff MD, MacCarthy T 2015 Overlapping hotspots in CDRs are critical sites for V region diversification. Proc Natl Acad Sci U S A 112:E728–E737. doi:10.1073/pnas.1500788112. 25646473 PMC4343087

[39] Halemano K, Guo K, Heilman KJ, Barrett BS, Smith DS, Hasenkrug KJ, Santiago ML 2014 Immunoglobulin somatic hypermutation by *Apobec3/Rfv3* during retroviral infection. Proc Natl Acad Sci U S A 111:7759–7764. doi:10.1073/pnas.1403361111. 24821801 PMC4040588

[40] MacMillan AL, Kohli RM, Ross SR 2013 APOBEC3 inhibition of mouse mammary tumor virus infection: the role of cytidine deamination versus inhibition of reverse transcription. J Virol 87:4808–4817. doi:10.1128/JVI.00112-13. 23449789 PMC3624289

[41] Mustafa F, Bhadra S, Johnston D, Lozano M, Dudley JP 2003 The type B leukemogenic virus truncated superantigen is dispensable for T-cell lymphomagenesis. J Virol 77:3866–3870. doi:10.1128/jvi.77.6.3866-3870.2003. 12610163 PMC149533

[42] Mertz JA, Mustafa F, Meyers S, Dudley JP 2001 Type B leukemogenic virus has a T-cell-specific enhancer that binds AML-1. J Virol 75:2174–2184. doi:10.1128/JVI.75.5.2174-2184.2001. 11160721 PMC114801

[43] Mertz JA, Kobayashi R, Dudley JP 2007 ALY is a common coactivator of RUNX1 and c-Myb on the type B leukemogenic virus enhancer. J Virol 81:3503–3513. doi:10.1128/JVI.02253-06. 17229714 PMC1866045

[44] Miller CL, Garner R, Paetkau V 1992 An activation-dependent, T-lymphocyte-specific transcriptional activator in the mouse mammary tumor virus env gene. Mol Cell Biol 12:3262–3272. doi:10.1128/mcb.12.7.3262. 1320198 PMC364540

[45] Broussard DR, Mertz JA, Lozano M, Dudley JP 2002 Selection for c-myc integration sites in polyclonal T-cell lymphomas. J Virol 76:2087–2099. doi:10.1128/jvi.76.5.2087-2099.2002. 11836386 PMC153816

[46] Nusse R, Varmus HE 1982 Many tumors induced by the mouse mammary tumor virus contain a provirus integrated in the same region of the host genome. Cell 31:99–109. doi:10.1016/0092-8674(82)90409-3. 6297757

[47] Ball JK, Arthur LO, Dekaban GA 1985 The involvement of a type-B retrovirus in the induction of thymic lymphomas. Virology 140:159–172. doi:10.1016/0042-6822(85)90455-6. 2981451

[48] Ball JK, Dekaban GA, McCarter JA, Loosmore SM 1983 Molecular biological characterization of a highly leukaemogenic virus isolated from the mouse. III. Identity with mouse mammary tumour virus. J Gen Virol 64:2177–2190. doi:10.1099/0022-1317-64-10-2177. 6311950

[49] Bhadra S, Lozano MM, Dudley JP 2005 Conversion of mouse mammary tumor virus to a lymphomagenic virus. J Virol 79:12592–12596. doi:10.1128/JVI.79.19.12592-12596.2005. 16160187 PMC1211542

[50] Suspène R, Rusniok C, Vartanian J-P, Wain-Hobson S 2006 Twin gradients in APOBEC3 edited HIV-1 DNA reflect the dynamics of lentiviral replication. Nucleic Acids Res 34:4677–4684. doi:10.1093/nar/gkl555. 16963778 PMC1635257

[51] Sheehy AM, Gaddis NC, Choi JD, Malim MH 2002 Isolation of a human gene that inhibits HIV-1 infection and is suppressed by the viral Vif protein. Nature 418:646–650. doi:10.1038/nature00939. 12167863

[52] Yu X, Yu Y, Liu B, Luo K, Kong W, Mao P, Yu X-F 2003 Induction of APOBEC3G ubiquitination and degradation by an HIV-1 Vif-Cul5-SCF complex. Science 302:1056–1060. doi:10.1126/science.1089591. 14564014

[53] Kobayashi M, Takaori-Kondo A, Miyauchi Y, Iwai K, Uchiyama T 2005 Ubiquitination of APOBEC3G by an HIV-1 Vif-Cullin5-Elongin B-Elongin C complex is essential for Vif function. J Biol Chem 280:18573–18578. doi:10.1074/jbc.C500082200. 15781449

[54] Hagen B, Kraase M, Indikova I, Indik S 2019 A high rate of polymerization during synthesis of mouse mammary tumor virus DNA alleviates hypermutation by APOBEC3 proteins. PLoS Pathog 15:e1007533. doi:10.1371/journal.ppat.1007533. 30768644 PMC6395001

[55] MacCarthy T, Kalis SL, Roa S, Pham P, Goodman MF, Scharff MD, Bergman A 2009 V-region mutation in vitro, in vivo, and in silico reveal the importance of the enzymatic properties of AID and the sequence environment. Proc Natl Acad Sci U S A 106:8629–8634. doi:10.1073/pnas.0903803106. 19443686 PMC2682541

[56] Golovkina TV, Jaffe AB, Ross SR 1994 Coexpression of exogenous and endogenous mouse mammary tumor virus RNA in vivo results in viral recombination and broadens the virus host range. J Virol 68:5019–5026. 8035502 10.1128/jvi.68.8.5019-5026.1994 PMC236444

[57] Golovkina TV, Piazzon I, Nepomnaschy I, Buggiano V, de Olano Vela M, Ross SR 1997 Generation of a tumorigenic milk-borne mouse mammary tumor virus by recombination between endogenous and exogenous viruses. J Virol 71:3895–3903. 9094666 10.1128/jvi.71.5.3895-3903.1997 PMC191541

[58] Xu L, Wrona TJ, Dudley JP 1996 Exogenous mouse mammary tumor virus (MMTV) infection induces endogenous MMTV sag expression. Virology 215:113–123. doi:10.1006/viro.1996.0014. 8560758

[59] Rassa JC, Meyers JL, Zhang Y, Kudaravalli R, Ross SR 2002 Murine retroviruses activate B cells via interaction with Toll-like receptor 4. Proc Natl Acad Sci U S A 99:2281–2286. doi:10.1073/pnas.042355399. 11854525 PMC122356

[60] Mehle A, Goncalves J, Santa-Marta M, McPike M, Gabuzda D 2004 Phosphorylation of a novel SOCS-box regulates assembly of the HIV-1 Vif-Cul5 complex that promotes APOBEC3G degradation. Genes Dev 18:2861–2866. doi:10.1101/gad.1249904. 15574592 PMC534646

[61] Byun H, Halani N, Gou Y, Nash AK, Lozano MM, Dudley JP 2012 Requirements for mouse mammary tumor virus Rem signal peptide processing and function. J Virol 86:214–225. doi:10.1128/JVI.06197-11. 22072771 PMC3255906

[62] Kao S, Khan MA, Miyagi E, Plishka R, Buckler-White A, Strebel K 2003 The human immunodeficiency virus type 1 Vif protein reduces intracellular expression and inhibits packaging of APOBEC3G (CEM15), a cellular inhibitor of virus infectivity. J Virol 77:11398–11407. doi:10.1128/jvi.77.21.11398-11407.2003. 14557625 PMC229358

[63] Alce TM, Popik W 2004 APOBEC3G is incorporated into virus-like particles by a direct interaction with HIV-1 Gag nucleocapsid protein. J Biol Chem 279:34083–34086. doi:10.1074/jbc.C400235200. 15215254

[64] Matreyek KA, Engelman A 2013 Viral and cellular requirements for the nuclear entry of retroviral preintegration nucleoprotein complexes. Viruses 5:2483–2511. doi:10.3390/v5102483. 24103892 PMC3814599

[65] MacDuff DA, Demorest ZL, Harris RS 2009 AID can restrict L1 retrotransposition suggesting a dual role in innate and adaptive immunity. Nucleic Acids Res 37:1854–1867. doi:10.1093/nar/gkp030. 19188259 PMC2665220

[66] Bekerman E, Jeon D, Ardolino M, Coscoy L 2013 A role for host activation-induced cytidine deaminase in innate immune defense against KSHV. PLoS Pathog 9:e1003748. doi:10.1371/journal.ppat.1003748. 24244169 PMC3820765

[67] Ichikawa HT, Sowden MP, Torelli AT, Bachl J, Huang P, Dance GSC, Marr SH, Robert J, Wedekind JE, Smith HC, Bottaro A 2006 Structural phylogenetic analysis of activation-induced deaminase function. J Immunol 177:355–361. doi:10.4049/jimmunol.177.1.355. 16785531

[68] Shinkura R, Ito S, Begum NA, Nagaoka H, Muramatsu M, Kinoshita K, Sakakibara Y, Hijikata H, Honjo T 2004 Separate domains of AID are required for somatic hypermutation and class-switch recombination. Nat Immunol 5:707–712. doi:10.1038/ni1086. 15195091

[69] Okazaki I, Kotani A, Honjo T 2007 Role of AID in tumorigenesis. Adv Immunol 94:245–273. doi:10.1016/S0065-2776(06)94008-5. 17560277

[70] Lu Z, Tsai AG, Akasaka T, Ohno H, Jiang Y, Melnick AM, Greisman HA, Lieber MR 2013 BCL6 breaks occur at different AID sequence motifs in Ig-BCL6 and non-Ig-BCL6 rearrangements. Blood 121:4551–4554. doi:10.1182/blood-2012-10-464958. 23476051 PMC3668488

[71] Mertz JA, Chadee AB, Byun H, Russell R, Dudley JP 2009 Mapping of the functional boundaries and secondary structure of the mouse mammary tumor virus Rem-responsive element. J Biol Chem 284:25642–25652. doi:10.1074/jbc.M109.012476. 19632991 PMC2757966

[72] Bhadra S, Lozano MM, Payne SM, Dudley JP 2006 Endogenous MMTV proviruses induce susceptibility to both viral and bacterial pathogens. PLoS Pathog 2:e128. doi:10.1371/journal.ppat.0020128. 17140288 PMC1665650

[73] Dudley J, Risser R 1984 Amplification and novel locations of endogenous mouse mammary tumor virus genomes in mouse T-cell lymphomas. J Virol 49:92–101. 6317898 10.1128/jvi.49.1.92-101.1984 PMC255429

[74] Rada C, Jarvis JM, Milstein C 2002 AID-GFP chimeric protein increases hypermutation of Ig genes with no evidence of nuclear localization. Proc Natl Acad Sci U S A 99:7003–7008. doi:10.1073/pnas.092160999. 12011459 PMC124518

[75] Ramiro AR, Jankovic M, Callen E, Difilippantonio S, Chen H-T, McBride KM, Eisenreich TR, Chen J, Dickins RA, Lowe SW, Nussenzweig A, Nussenzweig MC 2006 Role of genomic instability and p53 in AID-induced c-myc-Igh translocations. Nature 440:105–109. doi:10.1038/nature04495. 16400328 PMC4601098

[76] Mustafa F, Al Amri D, Al Ali F, Al Sari N, Al Suwaidi S, Jayanth P, Philips PS, Rizvi TA 2012 Sequences within both the 5’ UTR and Gag are required for optimal in vivo packaging and propagation of mouse mammary tumor virus (MMTV) genomic RNA. PLoS One 7:e47088. doi:10.1371/journal.pone.0047088. 23077548 PMC3473059

[77] Mariani R, Chen D, Schröfelbauer B, Navarro F, König R, Bollman B, Münk C, Nymark-McMahon H, Landau NR 2003 Species-specific exclusion of APOBEC3G from HIV-1 virions by Vif. Cell 114:21–31. doi:10.1016/s0092-8674(03)00515-4. 12859895

[78] Purdy A, Case L, Duvall M, Overstrom-Coleman M, Monnier N, Chervonsky A, Golovkina T 2003 Unique resistance of I/LnJ mice to a retrovirus is due to sustained interferon gamma-dependent production of virus-neutralizing antibodies. J Exp Med 197:233–243. doi:10.1084/jem.20021499. 12538662 PMC2193815

[79] Li H 2014 Toward better understanding of artifacts in variant calling from high-coverage samples. Bioinformatics 30:2843–2851. doi:10.1093/bioinformatics/btu356. 24974202 PMC4271055

[80] Li H, Handsaker B, Wysoker A, Fennell T, Ruan J, Homer N, Marth G, Abecasis G, Durbin R, 1000 Genome Project Data Processing Subgroup. 2009 The Sequence Alignment/Map format and SAMtools. Bioinformatics 25:2078–2079. doi:10.1093/bioinformatics/btp352. 19505943 PMC2723002

[81] Wickham H 2016 ggplot2: elegant graphics for data analysis, 2nd ed Springer, Cham, Switzerland.

