# Supplementary material for: A Protein Antagonist of Activation-Induced Cytidine Deaminase Encoded by a Complex Mouse Retrovirus
Source: mBio. 2019 Aug 13;10(4):e01678-19. doi: 10.1128/mBio.01678-19 (PMC6692512; doi:10.1128/mBio.01678-19)
Supplement: TABLE S1 [file mBio.01678-19-st001.docx]

| **Table S1. Reversion of the SD site in TBLV-SD proviruses detected by Illumina sequencing** | | | | | | | | | | | |
| --- | --- | --- | --- | --- | --- | --- | --- | --- | --- | --- | --- |
| **Position^1^** | **WT**  **Seq.^2^** | **SD**  **Seq.^3^** | **Averaged read counts from three different TBLV-WT or SD-induced tumors** | | | | | | | | |
|  |  |  | **WT** | **SD** | **WT** | **SD** | | **WT** | **SD** | **WT** | **SD** |
|  |  |  | **% A^4^** | | **% C^4^** | | | **% G^4^** | | **% T^4^** | |
| **7335** | G | G | 0.15 | 0.22 | 0.31 | | 0.71 | 99.43 | 98.90 | 0.11 | 0.16 |
| **7336** | G | G | 0.14 | 0.68 | 0.11 | | 0.12 | 99.59 | 98.93 | 0.16 | 0.27 |
| **7337** | G | **A**^5^ | 0.18 | 88.45 | 0.31 | | 0.73 | 99.32 | 10.64 | 0.20 | 0.18 |
| **7338** | G | **C**^5^ | 0.11 | 0.16 | 0.29 | | 89.42 | 99.50 | 10.31 | 0.11 | 0.11 |
| **7339** | T | T | 0.79 | 0.15 | 1.44 | | 0.72 | 2.68 | 0.80 | 95.10 | 98.33 |
| **7340** | G | **C**^5^ | 0.20 | 0.66 | 0.10 | | 89.10 | 99.37 | 9.90 | 0.33 | 0.34 |
| **7341** | A | **T**^5^ | 98.67 | 9.90 | 0.49 | | 1.03 | 0.69 | 0.27 | 0.14 | 88.79 |
| **7342** | G | **C**^5^ | 0.10 | 0.30 | 0.07 | | 89.04 | 99.54 | 9.96 | 0.29 | 0.70 |
| **7343** | T | **A**^5^ | 0.67 | 88.77 | 1.05 | | 1.05 | 1.29 | 0.36 | 96.99 | 9.83 |

^1^ Base position in the reference genome Accession: AF228552.1.

^2^ Sequence of the plus-strand TBLV-WT cloned provirus at positions 7335-7343 spanning the splice site.

^3^ Sequence of the plus-strand TBLV-SD cloned provirus at positions 7335-7343 spanning the splice site.

^4^ Average percentage read counts at each base (100 being the maximum) in proviruses from three WT or SD-induced BALB/c tumors (~20,000 reads/tumor or ~60,000 reads). Some variation is due to PCR error.

^5^ Bases in bold were mutated in the TBLV-SD provirus, but sequence variation (read counts much less than 100) at this position in averaged reads from tumors indicates SD reversion by recombination with endogenous *Mtv*s.
