# Supplementary material for: A Protein Antagonist of Activation-Induced Cytidine Deaminase Encoded by a Complex Mouse Retrovirus
Source: mBio. 2019 Aug 13;10(4):e01678-19. doi: 10.1128/mBio.01678-19 (PMC6692512; doi:10.1128/mBio.01678-19)
Supplement: TABLE S2 [file mBio.01678-19-st002.docx]

| **Table S2. Mutation Frequency in the *c-Myc* gene from TBLV-WT and TBLV-SD proviruses from thymic tumors by Sanger sequencing** | | | |
| --- | --- | --- | --- |
| **Mutation** | **TBLV-WT**  **Mutation Frequency^1^** | **TBLV-SD**  **Mutation Frequency^1^** | **Fold Increase** |
| G to A | 0.10 | 0.10 | 1.0 |
| A to G | 0.30 | 0.30 | 1.0 |
| C to T | 0.10 | 0.10 | 1.0 |
| T to C | 0.20 | 0.20 | 1.0 |
| Transversions | 0.10 | 0.02 | 0.2 |

^1^ Mutations/number of clones. Based on Sanger sequencing of 720 bp of the plus-strand of the *c-Myc* gene clones (~40) obtained from three independent BALB/c tumors induced by TBLV-WT or TBLV-SD.
